# Imaging the brain and vascular reactions to headache treatments: a systematic review

**DOI:** 10.1186/s10194-023-01590-5

**Published:** 2023-05-24

**Authors:** R. Messina, R. H. Christensen, I. Cetta, M. Ashina, M. Filippi

**Affiliations:** 1 Neuroimaging Research Unit, Division of Neuroscience and Neurology Unit, IRCCS San Raffaele Scientific Institute, Via Olgettina, 60, 20132 Milan, Italy; 2 Danish Headache Center, Department of Neurology, Rigshospitalet Glostrup, Glostrup, Denmark

**Keywords:** Headache, Neuroimaging, Migraine, Biomarkers, Secondary headaches

## Abstract

**Background:**

Neuroimaging studies have made an important contribution to our understanding of headache pathophysiology. This systematic review aims to provide a comprehensive overview and critical appraisal of mechanisms of actions of headache treatments and potential biomarkers of treatment response disclosed by imaging studies.

**Main body:**

We performed a systematic literature search on PubMed and Embase databases for imaging studies investigating central and vascular effects of pharmacological and non-pharmacological treatments used to abort and prevent headache attacks. Sixty-three studies were included in the final qualitative analysis. Of these, 54 investigated migraine patients, 4 cluster headache patients and 5 patients with medication overuse headache. Most studies used functional magnetic resonance imaging (MRI) (*n* = 33) or molecular imaging (*n* = 14). Eleven studies employed structural MRI and a few used arterial spin labeling (*n* = 3), magnetic resonance spectroscopy (*n* = 3) or magnetic resonance angiography (*n* = 2). Different imaging modalities were combined in eight studies.

Despite of the variety of imaging approaches and results, some findings were consistent. This systematic review suggests that triptans may cross the blood–brain barrier to some extent, though perhaps not sufficiently to alter the intracranial cerebral blood flow. Acupuncture in migraine, neuromodulation in migraine and cluster headache patients, and medication withdrawal in patients with medication overuse headache could promote headache improvement by reverting headache-affected pain processing brain areas. Yet, there is currently no clear evidence for where each treatment acts, and no firm imaging predictors of efficacy. This is mainly due to a scarcity of studies and heterogeneous treatment schemes, study designs, subjects, and imaging techniques. In addition, most studies used small sample sizes and inadequate statistical approaches, which precludes generalizable conclusions.

**Conclusion:**

Several aspects of headache treatments remain to be elucidated using imaging approaches, such as how pharmacological preventive therapies work, whether treatment-related brain changes may influence therapy effectiveness, and imaging biomarkers of clinical response. In the future, well-designed studies with homogeneous study populations, adequate sample sizes and statistical approaches are needed.

**Supplementary Information:**

The online version contains supplementary material available at 10.1186/s10194-023-01590-5.

## Background

In the last decades, the field of headache research has progressed significantly in part due to the use of brain imaging techniques. Neuroimaging provides a means to noninvasively capture central and vascular mechanisms underlying the pathophysiology of headache disorders. Evidence from molecular imaging techniques and magnetic resonance imaging (MRI) approaches support the involvement of the trigeminovascular system, brainstem, diencephalic, visual and pain processing cortical areas during the different phases of migraine [1]. Studies investigating patients suffering from trigeminal autonomic cephalalgias have shown significant activation of the hypothalamus and nociceptive brain areas during and outside the headache attacks [2]. Functional and structural alterations of cortical and subcortical areas responsible for the perception of the pain have also been revealed in patients with secondary headaches, like medication overuse and post-traumatic headache [3, 4].

Along with revealing important insights on the neurobiology of headache, neuroimaging techniques have deepened our comprehension of how acute and preventive headache treatments work [5]. The use of imaging techniques also has the potential to identify biomarkers for treatment response. However, a comprehensive overview of the mechanisms of action of headache treatments and possible predictors of clinical response disclosed by imaging studies, is missing. Furthermore, there is a need to identify gaps in the literature to develop robust imaging biomarkers of treatment effect that might guide future drug development.

This review provides a systematic and critical appraisal of imaging studies investigating brain and vascular changes associated with treatments used to abort and prevent headache attacks and exploring imaging predictors of patients´ response.

## Methods

In accordance with the Preferred Reporting Items for Systematic reviews and Meta-Analyses (PRISMA) guidelines, we performed a systematic literature search using the online PubMed and Embase databases. The used search string is reported in Supplementary Table 1.

The search was performed from the inception date up to 21 December 2022. Articles identified by this search strategy and judged relevant for the topic of the review were also selected.

Inclusion criteria for the search were as follows: original human research; molecular imaging studies; MRI studies; use of English language; studies including patients with primary and secondary headache disorders (migraine, trigeminal autonomic cephalalgias, tension-type headache, post-traumatic headache, medication overuse headache); studies investigating acute and preventive headache treatments including adult and/or pediatric and/or adolescents patients; cross-sectional studies exploring imaging predictors of patients´ response; longitudinal studies exploring central effects of treatments; studies investigating the brain and/or cephalic vascular system; studies including asymptomatic patients; studies performed during spontaneous and/or provoked headache attacks. Exclusion criteria for the search were as follows: conference abstracts; reviews; unpublished data; studies investigating the extracephalic vascular system; studies investigating central effects of acute and preventive headache treatments in healthy controls.

After checking for duplicates, studies obtained from the databases search were divided in three and two investigators (RM and RHC, RM and IC, RHC and IC) independently screened the title, abstract and full text of papers according to the pre-defined criteria. Any possible disagreements were resolved by discussion.

## Results

**Fig. 1:**
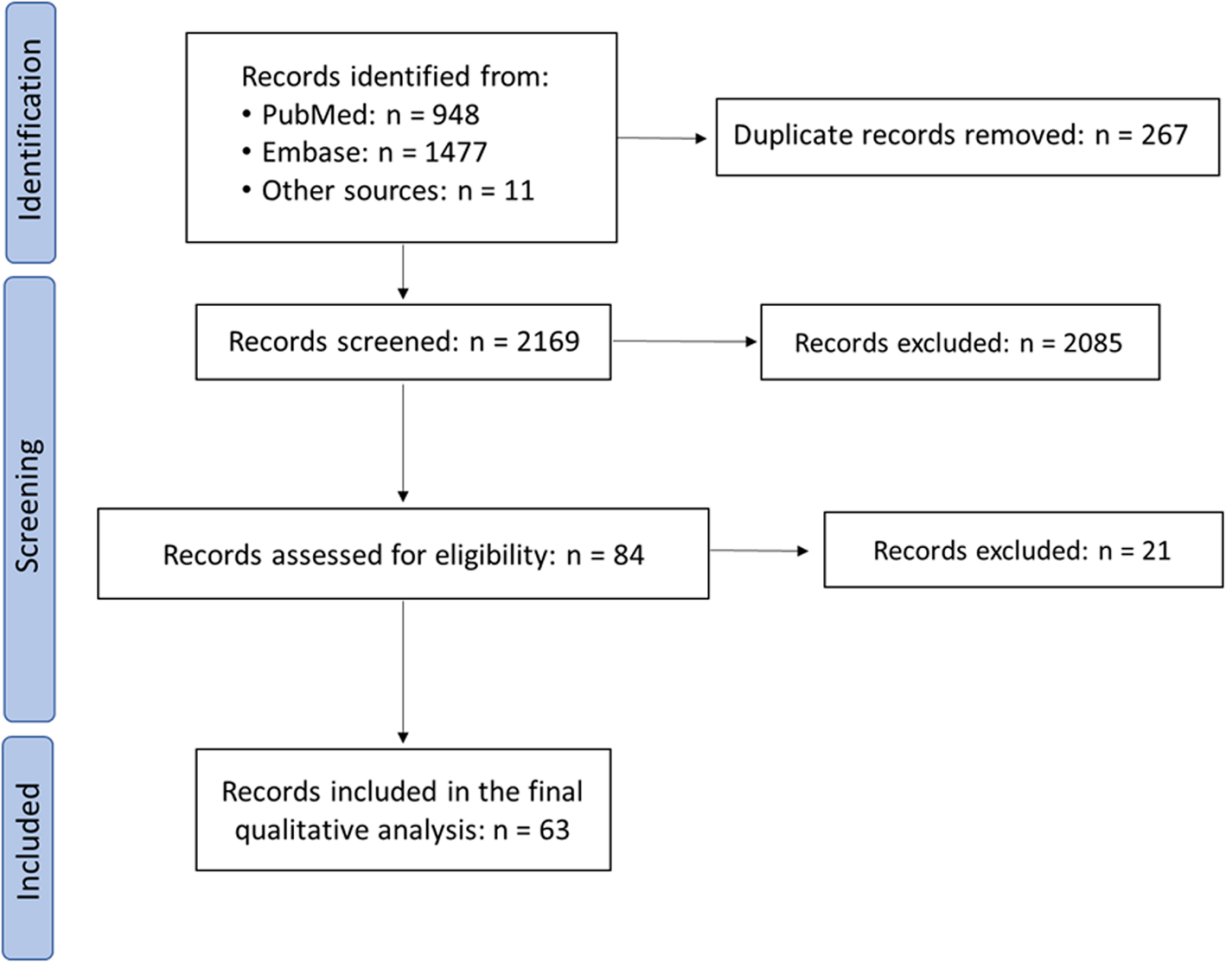
Flow chart of study selection

The database search identified 2425 records (PubMed: 948; Embase:1477). Eleven additional studies related to the topic were included. After duplicates were removed, the title and abstract of 2169 studies was screened yielding 84 articles for full-text screening. After full-text screening, 63 studies were included in the final qualitative analysis (Fig. 1).

Of the included studies, 54 (86%) investigated migraine patients, 4 (6%) cluster headache (CH) patients and 5 (8%) patients with medication overuse headache (MOH). No studies investigating central effects of acute and preventive treatments in patients with tension-type headache, hemicrania continua, paroxysmal hemicrania and post-traumatic headache were found.

Non-steroidal anti-inflammatory drugs (NSAIDs), triptans and ergotamine were examined in the included studies. While, no studies investigating steroids, indomethacin, oxygen, gepants or lasmitidan were found. Among preventive treatments, antiepileptics, calcium channel blocker, beta blockers and monoclonal antibodies targeting the calcitonin gene-related peptide (CGRP) were investigated. No studies examining gepants, antidepressants, anti-hypertensive or anti serotoninergic as prevention were found.

**Fig. 2:**
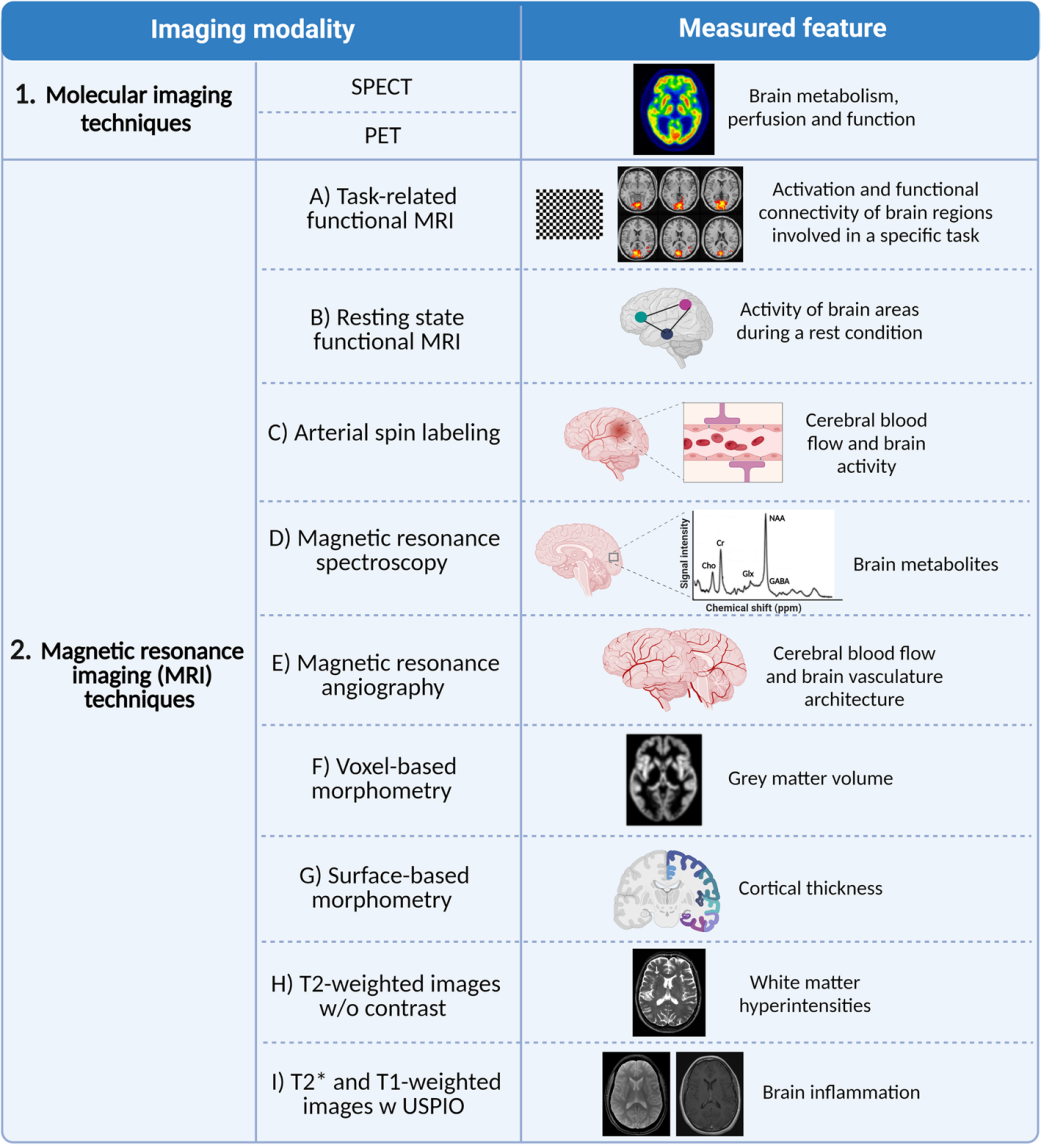
Imaging modalities employed by the included studies: 1) SPECT and PET are molecular imaging techniques that rely on the detection and quantification of rays released indirectly by radiolabelled molecules (tracers) injected into the body, thus providing information on the metabolism, perfusion and function of brain tissues [7]; 2A-B) Functional MRI (fMRI) techniques are based on the blood oxygenation level dependent mechanism. When a brain area is activated, the neuronal metabolism and regional cerebral blood flow (CBF) increase. The blood flow change is greater than the oxygen consumption, resulting in an increased ratio between the oxygenated and deoxygenated hemoglobin, which increase the MRI signal  [8]. fMRI approaches included task-related fMRI, which provide important information about the degree of activation and functional connectivity of brain regions that are involved in performing a specific task, and resting state (RS) fMRI that provide insight into the patterns of activity of brain networks or single brain areas during a rest condition [9]; 2C) Arterial Spin Labeling is a perfusion MRI technique that employs the arterial water to measure regional CBF changes associated with variations in regional neural activity [10]; 2D) Magnetic resonance spectroscopy is a non-invasive method that allows to identify and quantify metabolites present within a volume of interest based on the magnetic properties of their nuclei, mainly hydrogen and phosphorous. The main metabolites of interest are: N-acetylaspartate (NAA), a marker of neuronal integrity, choline (Cho), a marker of cellular membrane turnover, creatine (Cr), a marker of energy stores and the glutamate-glutamine and gamma-aminobutyric acid (Glx and GABA) neurotransmitters [11]; 2E) Magnetic resonance angiography is an approach that based on the magnetic properties of blood and surrounding tissues highlight the vasculature from the background without the use of contrast [12]; 2F-G) High resolution T1-weighted MRI with voxel-based (VBM) and surface-based morphometric (SBM)approaches provide information regarding the regional grey matter volume and cortical thickness; [13, 14] 2H) T2-weighted images without contrast can provide information regarding the presence of white matter hyperintensities. 2I) T2* and T1-weighted MRI with ultrasmall superparamagnetic iron oxide (USPIO), a cellular MR contrast agent, allows to investigate the macrophage-mediated inflammation [15]. Created by R.M. with BioRender.com

Figure 2 summarizes imaging modalities employed by the included studies. Fourteen studies applied molecular imaging approaches, including single-photon emission computerized tomography (SPECT) (*n* = 3) and positron emission tomography (PET) (*n* = 11). Six studies combined PET with MRI (*n* = 4) or computed tomography (*n*= 2) to increase the spatial resolution of the technique [6]. Distribution of MRI modalities used in the included studies was: 33 studies using functional MRI (fMRI) (9 task-related and 25 resting state (RS) fMRI studies); three studies using Arterial Spin Labeling (ASL); three studies using Magnetic Resonance Spectroscopy (MRS); two studies using MR angiography; 10 studies using high resolution T1-weighted (*n* = 8) or T2-weighted MRI without contrast (*n* = 2); one study using enhanced structural MRI during ultrasmall superparamagnetic iron oxide (USPIO) administration. Different imaging modalities were combined in eight studies.

**Fig. 3:**
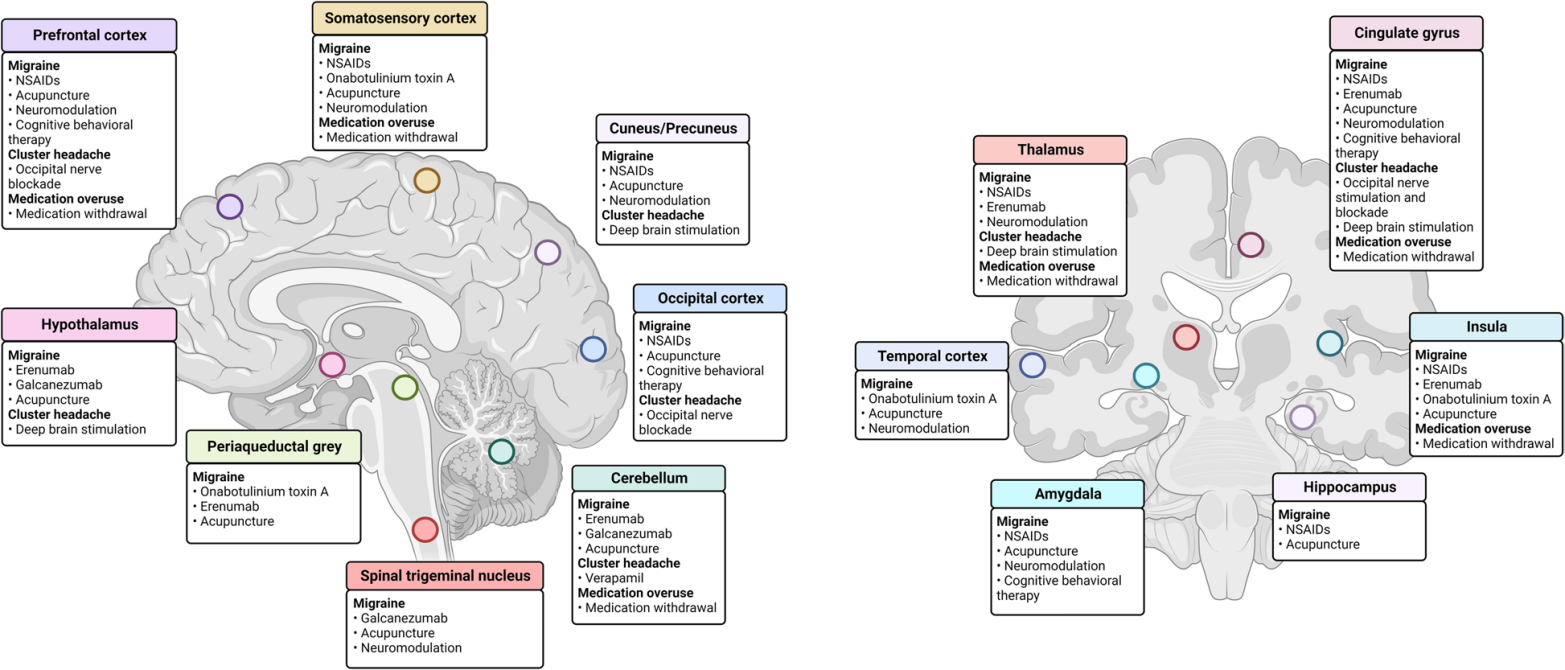
A schematic illustration of the main central areas targeted by treatments described in the included studies. Created by R.C.H. and I.C. with BioRender.com

For each individual study, study population and main results are presented in Tables 1, 2, 3 and 4 and Supplementary Tables 2, 3 and 4. Figure 3 outlines the main central structures targeted by treatments described in the included studies.

**Table 1:** Acute therapies for migraine

Reference	Population	Treatment	Imaging modality and timing of scans	Results	Limitations
Wei et al*.*, 2022 [16]	70 MO 33 HC	**Type:** NSAID **Duration:** 2 attacks treated within 3 months of scan **Response definition:** 50% or greater reduction in pain intensity from pre-treatment	**Modality:** RS fMRI **Time-point:** Pretreatment	**Baseline: Responders vs non-responders:** **↑** FC between visual and auditory network in responders **↓** FC between sensorimotor and visual network in responders	Type of NSAID not specified FC compared at p < 0.05, uncorrected for multiple comparison Overlap of regions belonging to distinct networks
Wei et al*.*, 2022 [17]	73 MO 33 HC	**Type:** NSAID **Duration:** 2 attacks treated within 3 months of scan **Response definition:** 50% or greater reduction in pain intensity between the pre-treatment level and 2 h after taking medication	**Modality:** RS fMRI and T1w MRI **Time-point:** Pretreatment	**Baseline: Responders vs non-responders:** **↑** FC between left amygdala and right superior frontal gyrus, left calcarine sulcus, left superior parietal gyrus and paracentral lobule in responders **↓** FC between left amygdala and ipsilateral caudate nucleus in responders No whole-brain GM volume differences	Type of NSAID not specified
Ahmed et al*.*, 2022 [18]	500 Mx (150 MA, 235 CM)	**Type:** Ibuprofen 200–400 mg **Duration:** At least 2 months **Response definition:** Pain freedom within 2 h in ≥ 4 of 5 attacks while insufficient responders achieved pain freedom in ≤ 3 of 5 attacks	**Modality:** T2w MRI **Time-point:** Pretreatment	**Baseline: Responders vs non-responders:** Acute treatment responders less frequently had WMHs, had fewer WMHs, and smaller WMHs	Only T2-w images were used to assess WMHs. FLAIR acquisition would also be recommended The same population was assessed for response to preventive treatments. However, the association between patients’ response to acute and preventive medications have not been examined
Asghar et al*.*, 2011 [19]	24 MO (12 received sumatriptan)	**Type:** Sumatriptan 6 mg s.c **Duration:** One administration	**Modality:** MR-angiography **Time-point:** Pretreatment, at follow-up during CGRP-induced migraine attack and 15 min after treatment	**Baseline-to-follow-up:** **↑** MMA and MCA circumference during CGRP-induced migraine attack compared to baseline, and specific to the attack side **↓** MMA circumference after sumatriptan compared to pretreatment. For patients with unilateral headache, this was only on the pain side No change in MCA circumference after sumatriptan	
Khan et al*.*, 2019 [20]	26 MO	**Type:** Sumatriptan 6 mg s.c **Duration:** One administration	**Modality:** MR-angiography **Time-point:** Pretreatment, at follow-up during cilostazol-induced migraine attack and one hour after treatment	**Baseline-to-follow-up:** **↑** Bilateral circumference of MCA, ICA_cerebral_, ICA_cavernous_, and ECA from baseline to early ictal scan. Increase in MMA circumference only on the pain side **↓** Bilateral circumference of MMA, ICA_cavernous_, ECA and STA after sumatriptan No reduction in MCA, ICA_cerebral_ or basilar artery	
Ferrari et al*.*, 1995 [21]	15 MO	**Type:** Sumatriptan 6 mg s.c **Duration:** One administration	**Modality:** Tc 99 m HM-PAO SPECT **Time-point:** Pretreatment during migraine attack and 3–45 min after treatment	**Baseline-to-follow-up:** Sumatriptan did not change rCBF during the attack phase on the headache or non-headache side in frontal, occipital, parietal, temporal, white matter, or cerebellar region of interests	
Friberg et al*.,* 1991 [22]	6 MA, 4 MO 18 HC	**Type:** Sumatriptan 2 mg i.v **Duration:** One administration for both patients and HC	**Modality:** Xe-133 SPECT w/ MCA TCD **Time-point:** Pre-treatment during migraine attack and at follow-up 30 min after treatment HC were studied at the same time-points as patients	**Baseline-to-follow-up: Patients and HC** Global and regional CBF in ROIs unchanged 30 min after sumatriptan infusion in both patients and HC Symptom relief 30 min after sumatriptan infusion in patients	MCA mean blood flow velocity likewise unchanged on TCD. Combined with unchanged CBF, this indicates no dilation of the MCA
Deen et al*.*, 2019 [23]	8 MO	**Type:** Sumatriptan 6 mg s.c **Duration:** One administration	**Modality:** [^11^C]AZ10419369 PET-MRI **Time-point:** Pre-treatments during cilostazol-induced migraine attack and at follow-up 43 min after treatment	**Baseline-to-follow-up:** **↓** 5-HT_1B_ binding after sumatriptan treatment of migraine attack in all brain regions examined (dorsofrontal and ventrolateral prefrontal, orbitofrontal, anterior cingulate, sensorimotor, and insular cortices as well as amygdala)	One tailed paired t-test between study days at p < 0.05, uncorrected for seven comparisons
Khan et al*.,* Cephalalgia 2019 [24]	28 MO (12 received sumatriptan) 4 HC	**Type:** Sumatriptan 6 mg s.c **Duration:** One administration	**Modality:** MRI with USPIO contrast **Time-point:** Single scan performed 24 h after treatment and after cilostazol-induced attacks HC underwent the same MRI protocol as patients	**Sumatriptan-treated vs untreated attacks: Patients** **↓** bilateral USPIO uptake in the ACA for patients who received sumatriptan compared to those who didn’t ↓ reduction of USPIO intake on the pain side compared to the non-pain side in patients treated with sumatriptan **Baseline-to-follow-up: HC** No significant differences in USPIO intake between the right and left side of the brain	Post-hoc analysis
Sakai et al*.,* Cephalalgia 2014 [25]	6 MO (interictal patients) 6 HC	**Type:** Eletriptan 40 mg **Duration:** One administration	**Modality:** α-[^11^C]MTrp PET-MRI **Time-point:** Pretreatment interictally and at follow-up one hour after treatment in both patients and HC	**Baseline: Migraine vs HC** No significant differences **Baseline-to-follow-up: Patients and HC** **↓** cerebral 5-HT synthesis in whole brain for migraine patients No changes after treatment in healthy controls	Migraine subjects were not ictal at baseline scan
Schankin et al*.*, 2016 [26]	6 Mx (4 MO, 2 MA) 6 HC	**Type:** Dihydro-ergotamine (11C labelled) **Duration:** One administration	**Modality:**^11^C-Dihydro-ergotamine PET MRI **Time-point:** Pretreatment and at follow-up during GTN-induced attacks concomitant with treatment HC underwent the same protocol as patients	**Baseline-to-follow-up: Patients and HC** No ^11^C-DHE binding in central regions of interest at baseline or post-GTN for migraine patients or healthy controls ^11^C-DHE Binding in regions outside the BBB (choroid plexus, pituitary fossa, venous sinuses, and facial tissue) for both groups at all scans	
Wu et al*.,* 2022 [27]	105 Mx (73 in testing and 32 in validation cohort)	**Type:** Sumatriptan (unspecified) **Response definition:** Decreased in headache severity from moderate or severe to none or mild within 2 h of sumatriptan intake in at least 2 out of 3 attacks	**Modality:** T1w MRI **Time-point:** Pretreatment	**Baseline: Responders vs non-responders:** **↑** Left hippocampus volume in sumatriptan responders in the testing cohort Left hippocampal volume greater than 4,032.6 mm^3^ differentiated responders from non-responders, in the validation cohort	20 regions of interest compared on both sides without correction for multiple comparisons Left hippocampal volume not different for responders compared to non-responders in validation cohort

^*11*^*C-DHE* 11-carbon dihydroergotamine, *ACA* Anterior cerebral artery, *BBB* Blood–brain barrier, *CGRP* Calcitonin gene-related peptide, *CM* Chronic migraine, *ECA* External cerebral artery, *FC* Functional connectivity, *FLAIR* Fluid-attenuated inversion recovery, *fMRI* functional magnetic resonance imaging, *GM* Grey matter, *GTN* Glyceryl trinitrate, *HC* Healthy control, *ICA*_*cavernous*_ Cavernous part of the internal carotid artery, *ICA*_*cerebral*_ Cerebral part of the internal carotid artery, *MA* Migraine with aura, *MCA* Middle cerebral artery, *MMA* Middle meningeal artery, *MO* Migraine without aura, *MRI* Magnetic resonance imaging, *Mx* Migraine with or without aura, *NSAID* Non-steroid anti-inflammatory drug, *PET* Positron emission tomography, *rCBF* regional cerebral blood flow, *RS* Resting-state, *SPECT* Single-photon emission computed tomography, *T1w* T1-weighted, *T2w* T2-weighted, *Tc 99 m HM-PAO* 99-technetium hexamethylpropylenamine oxime, *TCD* Transcranial doppler, *USPIO* Ultrasmall superparamagnetic iron oxide, *WMH* White-matter hyperintensities, *Xe-133* 133-xenon

**Table 2:** Pharmacological preventive treatments for migraine

Reference	Population	Treatment	Imaging modality and timing of scans	Results	Limitations
Chugani et al*.,* 1999 [28]	11 female Mx (5 studied after treatment) 8 female HC	**Type:** Propranolol (40 to 180 mg daily) or nadolol (20 to 40 mg daily) **Duration:** 12 weeks	**Modality:** [^**11**^C]AMT PET **Time-point:** Pretreatment and at follow-up after 12 weeks of treatment	**Baseline: Patients vs HC** Whole brain serotonin synthesis was higher in patients compared to HC **Baseline-to-follow-up: Patients** No difference in whole brain serotonin synthesis from baseline compared to after 12 weeks of treatment	Small sample size
Hebestreit et al*.*, 2017 [29]	19 Mx (4 MA, 6 CM) 26 HC	**Type:** Metoprolol 75 mg for patients Metoprolol 75 mg or placebo for HC **Duration:** 2 months minimum	**Modality:** Task-based fMRI w/ noxious trigeminal stimulus and visual stimulation **Time-point:** Pretreatment and at follow-up after minimum 2 months of treatment for patients HC underwent only one MRI after 50 min from drug administration	**Baseline-to-follow-up: Patients** No brain functional changes after treatment with metoprolol **↑** Hypothalamic activity after metoprolol treatment (*exploratory analysis*) Negative correlation between hypothalamic activity and reduction of headache days on metoprolol **Placebo vs Metoprolol in HC**: No significant functional brain differences	Exploratory analysis was uncorrected for multiple comparisons Single metoprolol or placebo administration in HC No placebo-controlled design for patients
Ahmed et al*.*, 2022 [18]	500 Mx (150 MA, 235 CM)	**Type:** Topiramate 2–200 mg **Duration:** At least 2 months **Response definition:** ≥ 50% reduction in the monthly headache days frequency compared to the baseline frequency	**Modality:** T2w MRI **Time-point:** Pretreatment	**Baseline: Responders vs non-responders:** Treatment responders less frequently had WMHs, had fewer and smaller WMHs	Only T2-w images were used to assess WMHs. FLAIR acquisition would also be recommended The same population was assessed for response to acute treatments. However, the association between patients’ response to acute and preventive medications have not been examined
Li et al*.*, 2018 [30]	14 Mx (13 MO, 1 MA)	**Type:** Levetiracetam 500 mg daily **Duration:** 12 weeks	**Modality:**^1^H-MRS for GABA **Time-point:** Pretreatment and at follow-up after 12 weeks of treatment	**Baseline-to-follow-up:** **↓** GABA levels in PCC after treatment No change in ACC/PFC GABA levels	Data available for 11 patients for the PCC and 8 for the ACC/PFC No strong evidence for levetiracetam as a migraine preventive medication Small sample size
Wöber et al*.,* 1994 [31]	11 Mx (6 responders, 5 non-responders) 21 HC	**Type:** Flunarizine 10 mg daily **Duration:** Ranged from 1 to 32 months **Response definition:** ≥ 50% reduction in migraine days	**Modality:**^123^I-Iodobenazamide SPECT **Time-point:** Single scan obtained after 1 to 32 months of treatment in patients HC underwent only one SPECT scan	**Responders vs non-responders** No difference in D2 receptor binding between responders and non-responders **Patients vs HC**: **↓** D2 receptor binding in flunarizine treated migraine patients compared to HC	Small sample size
Dominguez et al*.,* 2020 [32]	62 CM (47 responders to botox, 15 non-responders)	**Type:** Botox 155 IU every 12 weeks (PREEMPT protocol) **Duration:** 12 weeks **Response definition:** ≥ 50% reduction in frequency of headache	**Modality:** T2w MRI **Time-point:** Pretreatment	**Baseline: Responders vs non-responders** **↑** Iron accumulation in the globus pallidus and periaqueductal gray matter in non-responders compared to responders. Significant only for PAG after adjustment for age Differences in iron PAG deposits were associated with poor response to botox after adjustment for clinical and biochemical variables	No quantitative assessment of T2-w signal as a marker of iron accumulation
Hubbard et al*.*, 2016 [33]	23 CM (11 responder, 12 non-responders)	**Type:** Botox 150 IU every 12 weeks **Duration:** At least 12 weeks (at least 2 cycle of treatments) **Response definition:** ≥ 50% reduction in frequency of headache	**Modality:** T1w MRI and RS fMRI **Time-point:** Pretreatment	**Baseline: Responders vs non-responders** **↑** CTh of the right primary somatosensory cortex, anterior insula, left superior temporal gyrus and left inferior frontal gyrus (pars opercularis) in responders compared to non-responders **↓** FC between the right primary somatosensory cortex and left lateral occipital cortex and right dorsomedial prefrontal cortex, as well as between the left inferior frontal gyrus and left lateral occipital cortex and inferior supramarginal gyrus in responders *vs* non-responders	SBM not adjusted for gender Small sample size
Ziegler et al*.,* 2020 [34]	27 Mx (9 MA, 18 MO; 15 CM, 12 EM; 9 responders, 8 non-responders)	**Type:** Erenumab 70 mg every 4 weeks **Duration:** 3 months **Response definition:** ≥ 30% reduction in headache frequency after 3-month treatment	**Modality:** Task-based fMRI w/ noxious trigeminal stimulus and ASL **Time-point:** Pretreatment and at follow-up after 2–3 weeks of treatment	**Baseline-to-follow-up:** **↓** Activation in the thalamus, cerebellum, and several pain processing cortical areas after erenumab treatment The absolute reduction in headache days was correlated with reduced activity of the right putamen, hypothalamus, cerebellum, and thalamus, from baseline to follow-up, in patients within the same migraine phase at the two MRI time points (17 patients) **↓** FC between the hypothalamus and bilateral temporal lobe, bilateral cerebellum, left hippocampus, parahippocampus, fusiform gyrus, nucleus ruber and spinal trigeminal nucleus. **↑** FC between the right hypothalamus and the right anterior insula after treatment with erenumab No changes in rCBF **Baseline-to-follow-up: Responders vs non-responders:** **↓** Activation in the cerebellum, insula and hypothalamus in responders compared to non-responders (analyses including only the subgroup of patients within same migraine phase: 9 responders *vs* 8 non-responders)	Field of view optimized for brainstem imaging, so areas above the thalamus were not included Heterogenous sample size: patients with CM and EM; patients with MA and MO; patients taking other preventive medications; 10 patients having headache either at baseline or follow-up visit Results uncorrected for multiple comparisons Small sample size of subgroups included in the analysis responders *vs* non-responders
Basedau et al*.,* 2022 [35]	Patients treated with galcanezumab: 26 Mx (11 CM, 15 EM; 7 MA, 19 MO; 8 responders, 7 non-responders) Patients treated with erenumab: 17 Mx (same cohort included in Ziegleret al., 2020 [34]	**Type:** Galcanezumab 240 mg s.c. single dose Erenumab 140 mg every 4 weeks **Duration:** 3 months **Response definition:** ≥ 30% reduction in headache frequency after 3-month treatment	**Modality:** Task-based fMRI w/ painful thermal stimulation of left forearm and RS fMRI **Time-point:** Pretreatment and at follow-up after 8 weeks of treatment	**At follow-up:** **↓** FC of the spinal trigeminal nucleus with the hypothalamus and superior temporal gyrus, and increased FC with the cerebellum, middle temporal gyrus, and insula **Baseline-to-follow-up:** **↓** Activation in the right hypothalamus, right cerebellum, and cerebellar vermis after 2–3 weeks of treatments Correlation between activation in the spinal trigeminal nucleus at baseline and the absolute reduction in headache days No changes in rCBF **Baseline-to-follow-up: Responders vs non-responders:** **↓** Activation of brain areas including the inferior parietal, precentral, parahippocampal cortex and cerebellum in responders compared to non-responders (analyses including only the subgroup of patients within same migraine phase: 8 responders *vs* 7 non-responders) **Baseline-to-follow-up: erenumab vs galcanezumab:** Compared to erenumab, galcanezumab treatment reduced the activity of the pons, right substantia nigra, left thalamus, and right hypothalamus Compared to galcanezumab, erenumab treatment reduced the activity of the insula, thalamus, cerebellum, hippocampus, lingual gyrus, frontal, parietal and temporal brain areas Analyses were of only the subgroup of patients within the same migraine phase: 15 galcanezumab *vs* 17 erenumab	Results uncorrected for multiple comparisons Heterogenous sample size: patients with CM and EM; patients with MA and MO; patients taking other preventive medications; 11 patients having headache either at baseline or follow-up visit Small sample size of subgroups included in the analysis responders *vs* non-responders and galcanezumab *vs* erenumab
Schwedt et al*.,* 2022 [36]	32 Mx (21 CM, 11 EM; 18 responders, 14 non-responders)	**Type:** Erenumab 140 mg every 4 weeks **Duration:** 8 weeks **Response definition:** ≥ 50% reduction in the frequency of migraine days during week 5–8 of treatment	**Modality:** Task-based fMRI w/ painful thermal stimulation of left forearm and RS fMRI **Time-point:** Pretreatment and at follow-up after 8 weeks of treatment	**Baseline: Responders vs non-responders** **↓** Pain-induced response in the frontal supplemental motor region in responders compared to non-responders No RS FC differences **Follow-up: Responders vs non-responders** **↑** Pain-induced response in the left middle and posterior cingulate cortex, right putamen and periaqueductal gray matter in responders compared to non-responders **↑** Global network efficiency in responders compared to non-responders **↑** RS FC of the hypothalamus, fronto-parietal and temporal brain regions in responders compared to non-responders, as well as widespread differences between responders and non-responders in several graph theory metrics	Analyses uncorrected for multiple comparisons Heterogenous sample size: patients with CM and EM; 19 patients with MOH; patients taking other preventive medications; patients having headache either at baseline or follow-up visit No information regarding the presence of aura
Peek et al*.,* 2021 [37]	18 CM (8 treated with botox and 10 treated with erenumab)	**Type:** Botox 155 IU every 3 months (PREEMPT protocol) Or Erenumab 70 or 140 mg s.c. monthly **Duration:** 3 months	**Modality:** MRS measuring GABA and Glx levels in ACC and PCC **Time-point:** Pretreatment and at follow-up after 3 months of treatment	**Baseline-to-follow-up:** In the mixed treatment group, increased GABA levels in the ACC correlated with decreased migraine frequency, HIT-6, and MIDAS scores after treatments In post-hoc analysis, greater increase in ACC GABA levels and decrease in headache frequency and HIT-6 scores in patients receiving erenumab compared to those treated with botox	Mixed treatment group which did not differentiate effects of botox and erenumab for all comparisons Small sample size of subgroups of patients
Newman-Norlund et al*.,* 2020 [38]	12 CM w/ MOH	**Type:** Spheno-palatine ganglion block with nasal bupivacaine 0.5% **Duration:** 6 weeks (12 treatments)	**Modality:** T1w MRI **Time-point:** Pretreatment and at follow-up after 6 weeks of treatment	**Baseline to follow-up** **↑** Volume of left nucleus accumbens after treatment **↓** Volume of right hippocampus and pallidum, after treatment **↓** CTh of the left temporal pole and lateral occipital-temporal sulcus, after treatment	Statistical approach not described in detail
Krebs et al*.*, 2018 [39]	10 CM w/ MOH	**Type:** Spheno-palatine ganglion block with nasal bupivacaine 0.5% **Duration:** 6 weeks (12 treatments)	**Modality:** RS fMRI **Time-point:** Pretreatment and at follow-up after 6 weeks of treatment	**Baseline to follow-up** **Salience network:** **↑** FC between the left anterior prefrontal cortex and bilateral orbitofrontal insula, ventral striatum, right supplementary motor area and dorsal prefrontal cortex **↑** FC between the right ventral tegmental area/substantia nigra and left dorsolateral prefrontal cortex and right temporal pole **↑** FC between the left superior temporal cortex and right supramarginal gyrus **Executive network** **↑** FC between the left dorsolateral prefrontal cortex and right dorsolateral prefrontal cortex, ventrolateral prefrontal cortex, right anterior thalamus, and caudate nucleus **↑** FC between the left dorsal prefrontal cortex and right anterior thalamus, and caudate nucleus	Unclear whether the study used an unadjusted t-test or a permutation test

*[*^***11***^*C]AMT* Alpha-11C-methyl-L-tryptophan, *1H-MRS* Proton magnetic resonance spectroscopy, *ACC* Anterior cingulate cortex, *ASL* Arterial spin labeling, *CM* Chronic migraine, *CTh* Cortical thickness, *EM* Episodic migraine, *FC* Functional connectivity, *FLAIR* Fluid attenuated inversion recovery, *fMRI* functional magnetic resonance imaging, *GABA* Gamma amino butyrate, *HC* Healthy control, *IU* International units, *MA* Migraine with aura, *MO* Migraine without aura, *MOH* Medication-overuse headache, *MRI* Magnetic resonance imaging, *Mx* Migraine with or without aura, *PAG* Periaqueductal grey, *PCC* Posterior cingulate cortex, *PET* Positron emission tomography, *PFC* Prefrontal cortex, *PREEMPT* Phase III Research Evaluating Migraine Prophylaxis Therapy, *rCBF* regional cerebral blood flow, *RS* Resting state, SBM Surface-based morphometry, *T1w* T1-weighted, *T2w* T2-weighted, *WMH* white matter hyperintensities

**Table 3:** Preventive treatments for cluster headache

Reference	Population	Treatment	Imaging modality and timing of scans	Results	Limitations
May et al*.,* 2006 [40]	10 CCH	**Type:** Hypothalamic DBS stimulation **Duration:** 60 s of stimulation	**Modality:** H_2_^15^O-PET **Time-point:** Single-scan obtained with and without DBS stimulation	**Stimulation vs no stimulation:** **↑** Activation during stimulation in the ipsilateral hypothalamus, thalamus, trigeminal nucleus and ganglion, precuneus, somatosensory, and anterior cingulate cortex **↓** Activation during stimulation in the bilateral middle temporal gyrus, posterior cingulate cortex, inferior temporal gyrus, and contralateral anterior insula	Uncorrected results and small volume correction for a priori selected ROIs Five patients were taking other preventive medications No information regarding the cluster headache phase (in bout or out of bout) and the presence of headache the day of the PET scan
Magis et al*.,* 2011 [41]	10 CCH (drug-resistant) (7 responders and 3 non-responders) 39 HC	**Type:** Occipital nerve stimulation **Duration:** 6–30 months	**Modality:**^18^FDG PET **Time-point:** Before treatment, after 1 month and after 6 months of treatment (6 CH) After 24 to 30 months after treatment (4 CH) HC underwent only one PET scan	**CCH vs HC:** **↑** metabolism in the ACC, left hypothalamus, left pulvinar, left visual cortex, cerebellum and brain stem in CCH compared to HC **↓** metabolism in bilateral sensorimotor areas in CCH compared to HC **Baseline-to-follow-up (after 1 month):** No significant changes **Baseline-to-follow-up (after ≥ 6 months):** **↓** metabolism in bilateral cingulate cortex, left visual cortex, pulvinar, midbrain and pons, **↑** metabolism in bilateral sensorimotor areas **Follow-up: Responders vs non-responders** **↑** metabolism in left perigenual ACC in responders compared to non-responders at follow-up	Small sample size
Medina et al*.,* 2021 [42]	18 CH 7 HC	**Type:** Greater occipital nerve blockade with methylprednisolone 80 mg and 2 ml of lidocaine 2% **Duration:** Single blockade	**Modality:** ASL **Time-point:** Before and 7 days after treatment HC underwent only one MRI sca	**Baseline: Patients vs HC** **↑** rCBF in the cerebellum and left hippocampus in patients compared to HC **↓** rCBF in the right orbitofrontal cortex, rostral anterior insula and middle temporal gyrus in patients compared to HC **Baseline-to-follow-up:** **↑** rCBF in the right secondary visual cortices **↓** rCBF in the left medial temporal gyrus, cerebellum, caudate and putamen **Baseline: Responders vs non-responders** **↑** rCBF in the right lateral occipital cortex and left medial prefrontal cortex in responders compared to non-responders **↓** rCBF in the right posterior cingulate gyrus in responders compared to non-responders **Follow-up: Responders vs non-responders** **↓** rCBF in the left middle temporal cortex in responders compared to non-responders	Uncorrected results and small volume correction for a priori selected regions of interest
Tso et al*.,* 2021 [43]	194 CH (105 non-responders, 89 responders)	**Type:** Verapamil (variable dosage) **Duration:** ≥ 3 months	**Modality:** T1w MRI **Time-point:** Before treatments	**Baseline: Responders vs non-responders** **↑** GM volume of the cerebellar vermis (FWE-corrected results) and bilateral cerebellar lobule VI (uncorrected results) in non-responders compared to responders A supervise machine learning model discriminated responders from non-responders with an accuracy of 66% based on clinical data, and of 68% based on combined clinical and imaging data	Patients with probable and post-traumatic cluster-headache included Data retrieved retrospectively from medical records Missing data on bout of patients No harmonization efforts to adjust for the use of different scanners

*18-FDG* 18-flourodeoxyglucose, *ACC* Anterior cingulate cortex, *ASL* Arterial spin labeling, *CCH* Chronic cluster headache, *CH* Cluster headache, *DBS* Deep brain stimulation, ^*18*^*FDG* 18-fluorodeoxyglucose, *FWE* Family-wise error, *HC* Healthy controls, *PET* Positron emission tomography, *rCBF* regional cerebral blood flow, *ROI* Region of interest

**Table 4:** Medication withdrawal in patients with medication overuse headache

Reference	Population	Treatment	Imaging modality and timing of scans	Results	Limitations
Fumal et al*.*, 2006 [44]	16 CM w/ MOH 68 HC	**Type:** Medication withdrawal	**Modality: **^**18**^ FDG PET-MRI **Time-point:** Pre-withdrawal and at follow-up 3 weeks after withdrawal for patients HC underwent only one scan	**Baseline: HC *vs* MOH** **↓** Metabolism in bilateral thalamus, insula/ventral striatum, orbitofrontal cortex, and right posterior parietal cortex in MOH compared to HC **↑** Metabolism in the cerebellar vermis in patients compared to HC **Baseline to follow-up:** **↑** Metabolism in MOH in the insula, thalamus, parietal cortex, and cerebellum after withdrawal **↓** Metabolism in the orbitofrontal cortex persisted and was more pronounced after withdrawal in patients compared to HC	Uncorrected results and small volume correction for a priori selected ROIs Smaller sample of patients compared to the sample of HC No information regarding use of acute medications before the pre-withdrawal scan
Ferraro et al*.,* 2021 [45]	8 MOH without medication withdrawal (MOH) 8 MOH with medication withdrawal (D-MOH) 8 CM 8 HC	**Type:** Medication withdrawal	**Modality:** Task-based fMRI w/ decision making **Time-point:** D-MOH had the MRI 6 months after withdrawal MOH, HC and CM underwent one MRI scan	**Baseline:** **↑** Activation in the bilateral ventromedial prefrontal cortex and precuneus in MOH compared to HC and D-MOH **↓** Activation in midbrain regions (substantia nigra/ventral tegmental area) in MOH compared to HC and CM patients **↓** Activation in midbrain regions (substantia nigra/ventral tegmental area) in D-MOH compared to HC No midbrain differences between MOH and D-MOH	Decision making involved a monetary gamble with unknown relation to medication intake
Mehnert et al*.,* 2018 [46]	18 MOH 18 HC	**Type:** Medication withdrawal	**Modality:** Task-based fMRI w/ noxious trigeminal stimulus and T1w MRI **Time-point:** Pre-withdrawal and at follow-up 8 weeks after withdrawal HC underwent the same protocol of patients	**fMRI—Baseline to follow-up:** **↑** Activation of the left spinal trigeminal nucleus, right operculum, and posterior insula in MOH patients after withdrawal **↑** FC during nociception of the right medial orbital gyrus with the right spinal trigeminal nucleus and bilateral cerebellum in MOH patients after withdrawal No significant longitudinal functional differences between patients and controls **VBM—Baseline to follow-up:** **↓** GM volume of the left cuneus, superior temporal gyrus, putamen, and cerebellum in patients after withdrawal No significant longitudinal GM volume differences between patients and controls The absolute reduction in headache days was correlated with GM volume of right medial orbital gyrus in MOH	Uncorrected results with following application of FWE small volume correction No information regarding use of acute medications before the pre-withdrawal scan 11 patients started using preventive drugs for migraine during the study
Ferraro et al*.,* 2012 [47]	9 MOH (all female) 9 HC (all female)	**Type:** Medication withdrawal	**Modality:** Task-based fMRI w/ painful stimulation of the left hand **Time-point:** Pre-withdrawal and at follow-up 6 months after withdrawal HC underwent only one MRI	**Baseline: HC vs MOH** **↑** Pain-related activation in primary somatosensory cortex, inferior parietal cortex, and supramarginal gyrus in MOH compared to HC **Follow-up: HC vs MOH** No difference in MOH compared to HC at follow-up	Small sample size Uncorrected results with following application of FWE small volume correction Extracephalic nociceptive stimulation
Riederer et al*.,* 2013 [48]	22 MOH (11 responders, 11 non-responders)	**Type:** Medication withdrawal	**Modality:** T1w MRI **Time-point:** Pre-withdrawal and at follow-up 3 months after withdrawal	**Baseline: Responders vs non-responders** **↑** GM volume of the right orbitofrontal cortex in responders compared to non-responders Positive correlation between GM volume of the orbitofrontal cortex and patients´ treatment response **Baseline to follow-up:** **↓** GM volume of the midbrain (PAG and reticular formation) in responders No significant longitudinal GM volume changes in non-responders	Small sample size of subgroups of responders and non-responders

^*18*^*FDG*^18^Flourodeoxyglucose, *CM* Chronic migraine, *FC* Functional connectivity, *fMRI* Functional magnetic resonance imaging, *FWE* Family-wise error, *GM* Grey matter, *HC* Healthy control, *MOH* Medication-overuse headache, *MRI* Magnetic resonance imaging, *PAG* Periaqueductal grey, *PET* Positron emission tomography, *ROI* Region of interest, *T1w* T1-weighted, *VBM* Voxel based morphometry

### Migraine

#### Acute treatments

##### Non-steroidal anti-inflammatory drugs

Three fMRI studies [16–18] have examined imaging predictors of response to NSAIDs. The first of these used pre-treatment RS fMRI to predict the response to NSAIDs [16]. In 70 patients without aura, they found that the visual network in responders had decreased functional connectivity (FC) with the somatosensory network and increased FC with the auditory network, compared to non-responders. In addition, a support vector machine model based on pre-treatment FC reported a 93% accuracy in predicting responders. In another study [17] based on the same cohort and focused on the FC of the left amygdala, responders had increased FC with the left calcarine, superior frontal, and parietal areas, as well as a decreased FC with the ipsilateral caudate nucleus, compared to non-responders. Pre-treatment RS FC of the amygdala with the caudate, visual and frontoparietal areas predicted patients’ response with an accuracy of 89%. Finally, one study [18] examined whether white matter hyperintensities (WMHs) could predict a consistent response to ibuprofen, defined as pain freedom within two hours in at least four of five treated attacks. Using T2 weighted imaging, the study examined 500 patients with migraine who treated their attacks with ibuprofen 200–400 mg. The study found that the 244 responders less frequently had WMHs, and that their WMHs were of a smaller size and diameter, than the 256 non-responders.

##### Triptans and ergotamines

Two early SPECT studies with Tc-99 m-HMPAO or Xe-133 [21, 22] demonstrated that treatment of the migraine attack with subcoutanoues sumatriptan was not associated with regional cerebral blood flow (CBF) changes. Similar findigs were found by two later MR angiography studies demonstrating that sumatriptan constricts extracerebral arteries such as the superficial temporal and middle meningeal arteries, but not intracerebral arteries [19, 20].

In one PET study using the 5-HT_1B_ receptor radioligand [^11^C]AZ10419369, eight patients with migraine without aura were examined during cilostazol-induced migraine attacks before and after receiving subcutaneous sumatriptan 6 mg [23]. Sumatriptan reduced serotonin receptor binding in pain modulating regions, including frontal areas, sensorimotor cortex, insula, and amygdala, by 16.0%. Another study [25] examined the effects of eletriptan on central serotonin synthesis in six participants with migraine without aura and six healthy controls using PET with tracer α-[^11^C]MTrp, a surrogate marker of cerebral 5-HT synthesis. In patients with migraine, eletriptan reduced the rate of 5-HT synthesis in the entire brain, whereas no change occurred in healthy controls.

There is also evidence showing that in 12 migraine patients examined by USPIO-enhanced MRI sumatriptan attenuated the uptake of USPIO in the anterior cerebral artery perfusion territory after cilostazol induced attack. USPIO uptake may reflect activated macrophages or extravasation, but the finding should be interpreted with caution due to its exploratory nature [24].

One study [27] also explored morphometric brain features associated with a good triptan response, showing a lower volume of the left hippocampus in sumatriptan responders compared to non-responders. Even so, sumatriptan response was defined retrospectively and the between-group comparison was not adjusted for age, gender, or total intracranial volume, which may all influence hippocampal volume. Likewise, the analysis was not corrected for multiple comparisons.

Only one study explored whether dihydroergotamine (DHE) has central effects. Six patients with migraine without aura received ^11^C-DHE before and after administration of nitroglycerin (GTN) to provoke a migraine attack. At PET-MRI before and 3 h after GTN infusion, ^11^C-DHE did not pass the BBB [26].

### Preventive treatments

#### Pharmacological approaches

##### Beta-blockers: Propranolol, nadolol and metoprolol

Two studies have examined whether beta-blockers caused cerebral changes, when used for migraine prevention. The first PET study [28] compared whole-brain serotonin synthesis before and after 12 weeks of propranolol or nadolol treatment in five migraine patients, using the ^11^C-AMT tracer. The study found that beta-blockers did not change whole-brain serotonin synthesis. In another study [29], Hebestreit et al*.* examined changes of task-based fMRI in response to trigeminal painful stimulation before and after at least 2 months of treatment with metoprolol 75 mg in 19 patients with migraine. The study found no significant functional brain changes after treatment. When performing an uncorrected exploratory analysis, metoprolol increased the hypothalamic BOLD response after treatment. However, the hypothalamic BOLD response correlated negatively with the reduction in headache days at follow-up. This is difficult to reconcile with a treatment effect.

##### Antiepileptic medication: Topiramate and levetiracetam

Ahmed and colleagues [18] sought to predict the efficacy of topiramate in migraine patients based on WMHs. The study enrolled 500 patients who underwent T2-weighted MRI prior to treatment with 2–200 mg topiramate for at least 2 months. The same cohort was investigated for patients’ response to Ibuprofen 200–400 mg. Like acute treatments, compared to non-responders, responders to topiramate less frequently had WMHs, and the WMHs were fewer and of smaller diameter. These findings should be replicated in a separate cohort.

Although there is no strong evidence supporting the superiority of levetiracetam over placebo and topiramate for migraine prevention [49], one study [30] examined changes in GABA concentration with MRS before and after 12 weeks of treatment with levetiracetam. This study found decrease GABA levels in the posterior cingulate cortex (PCC) after treatment, whereas anterior cingulate cortex (ACC) and prefrontal cortex levels were unchanged. The PCC is activated during pain, but whether the changes were associated with the treatment response was not examined.

##### Calcium channel blocker: Flunarizine

One study [31] examined whether differences in D2 receptor occupancy might affect the flunarizine response, using the D2 receptor ligand ^123^I-Iodobenzamide. The study found no differences in receptor binding between six responders and five non-responders. However, flunarizine still decreased dopamine binding in treated migraine patients compared to untreated healthy controls, suggesting that flunarizine does bind central D2 receptors, but other receptors or channels could mediate the migraine preventive effect.

##### Botulinum toxin

Dominguez et al*.* [32] examined whether iron deposition in subcortical structures could predict botox treatment response in chronic migraine. This study found a decreased T2-weighted signal in the periaqueductal grey (PAG) in 47 responders compared to 15 non-responders, suggesting increased iron accumulation in responders. However, it should be noted that the T2 weighted signal is not specific for iron accumulation. Another study [33] examined whether pre-treatment cortical structure and RS FC patterns distinguished botox responders from non-responders. The study found increased cortical thickness in several pain relevant areas, including the right primary somatosensory cortex, anterior insula and left inferior frontal gyrus, in responders compared to non-responders. Further examining the FC of these regions, compared to non-responders, responders showed an altered functional interaction between fronto-parietal pain processing areas and occipital regions implicated in visual processing.

##### Anti-CGRP monoclonal antibodies

So far, three MRI studies [34–36] have examined brain functional changes after treatment with anti-CGRP monoclonal antibodies (mAbs). Two studies [34, 35] used task-based fMRI with noxious trigeminal stimulation and ASL to investigate brain functional changes after 2–3 weeks of galcanezumab, a mAb targeting the CGRP ligand, and erenumab, a mAb targeting the CGRP receptor. In 27 patients, galcanezumab reduced the response to trigeminal stimulation in the right hypothalamus and cerebellum, whereas erenumab reduced the response in both cerebellar hemispheres, the left operculum, right thalamus, middle temporal, and lingual cortex in 26 patients. Comparing galcanezumab to erenumab, it has been shown that the two mAbs decreased the acitivity of different brain areas involved in nociceptive activity [35]. Neither erenumab nor galcanezumab changed the regional CBF. These studies explored also imaging features associated with patients’ response after 3 months of treatment. Galcanezumab treatment decreased the activity of the cerebellum, insula, and hypothalamus in responders compared to non-responders, while treatment with erenumab decreased the activity of many areas, including the parahippocampus, cerebellum, inferior parietal, and precentral cortex. The absolute reduction in monthly headache days correlated with higher pretreatment activity of the spinal trigeminal nucleus for galcanezumab, and with the decreased activity of the right putamen, hypothalamus, cerebellum, and thalamus observed after erenumab treatment.

Another study [36] examined functional changes after 2-month treatment with erenumab in 32 patients with migraine, using RS fMRI and fMRI during extracranial nociceptive stimulation. At follow-up, when compared to 14 non-responders, 18 responders had a greater pain-induced response in the left cingulate cortex, PAG and right putamen, as well as increased RS FC of the hypothalamus, fronto-parietal and temporal brain regions. At baseline, responders were distinguished by a decreased activity in the frontal supplemental motor areas in response to painful stimulation compared to non-responders. Finally, one study [37] used MRS to examine changes in ACC and PCC levels of GABA and glutamate. The primary analysis used a mixed population of patients receiving botox and erenumab, precluding firm findings regarding either dug, but a post-hoc analysis reported that the 18 patients who received erenumab had a greater increase in the GABA levels of the ACC, compared to the 10 patients who received botox.

##### Sphenopalatine ganglion block with local anesthetics

Two studies [38, 39] examined morphometric and functional brain changes after nasal-bupivicaine sphenopalatine ganglion blockade in patients with chronic migraine and MOH. Six weeks after twice weekly treatment, the studies reported an increased volume of the left nucleus accumbens, a decreased volume of the right hippocampus and pallidum, and decreased cortical thickness of the left temporal pole and lateral occipital-temporal sulcus cortex, as well as an altered FC of several regions of the salience and executive networks. However, these studies did not describe their statistical approach in detail, making the findings difficult to interpret.

#### Non-pharmacological approaches

##### Acupuncture

Of the reviewed studies, 17 (27%) explored functional and structural brain changes associated with acupuncture treatment. In the included studies, the acupoints selected varied greatly. The duration of each session ranged from 1 up to 30 min, the number of treatments per weeks was inconstant and the treatment period could range from 4 to 16 weeks. Sham acupuncture including inactive acupoints was used as a placebo control only in some studies.

Using RS fMRI, many studies [50–57] demonstrated that, compared to controls, migraine patients experienced widespread functional alterations in brain areas implicated in the processing of the sensory-discriminative, cognitive, and emotional aspects of pain, which were reverted after acupuncture treatment.

A few studies [52, 53, 57, 58] explored whether acupuncture-related functional brain changes were associated with patients´ improvement after treatment, showing an association between changes in brain activity and changes in the severity and frequency of migraine attacks.

Tian and colleagues [50] explored FC patterns associated with a good response to acupuncture, defined as at least 30% reduction in headache intensity or migraine attack frequency, showing that, compared to 29 patients who were non-responders, 19 responders had greater increases of thalamic FC after 4 weeks of treatment. Acupuncture-related thalamic changes have also been described by Gu et al*.* [59], who, using MRS, showed increased NAA/Cr ratio but unchanged Cho/Cr ratio in the thalamus in patients treated with acupuncture for nine weeks. An increase in the NAA/Cr ratio may reflect higher thalamic neuronal activity and energy metabolism as a result of acupuncture treatment. However, the study did not provide information regarding pre-treatment thalamic metabolism in migraine patients, thus precluding firm conclusions. It should be noted that, these studies did not include a sham group.

Recent studies [60–65] investigated neural changes associated with acupuncture comparing the effect of verum acupuncture to sham acupuncture, which included inactive acupoints. RS fMRI studies [60–62] described more extensive changes in the function of pain modulatory brain areas in patients receiving verum acupuncture compared to those treated with sham treatment. The study conducted by Li and colleagues [65] showed that only treatment with verum acupuncture could normalize the lower activity of the rostral ventromedial medulla revealed in migraine patients compared to controls before acupuncture initiation. The rostral ventromedial medulla is a pivotal area of the descending pain inhibitory system [66].

Two PET studies [63, 64] examined a small sample of patients with migraine demonstrating that 30 min of verum electro-acupuncture stimulation induced broad modifications in brain metabolism compared to sham stimulation.

Besides fMRI and molecular imaging, a recent study [67] aimed to explore the value of grey matter (GM) volume in predicting migraine patients´ response to acupuncture. Using a machine learning approach, the study showed that a predictive model including the GM volume of the calcarine cortex, precuneus, cuneus, temporal, frontal and parietal brain areas could discriminate responders from non-responders with an accuracy of 83%. This study also showed that, compared to non-responders, responders to 4-week of acupuncture treatment developed an increased GM volume of the left cuneus after treatment. However, these results should be validated in a different cohort.

##### Non-invasive and invasive neuromodulation

Some studies have explored whether central effects could occur secondary to non-invasive neuromodulation approaches. The study conducted by Russo and colleagues [68] showed an increased activation of the right ACC during trigeminal heat stimulation in 16 migraine patients compared to 16 age and sex-matched healthy controls. In migraine patients treated for two months with external trigeminal stimulation (eTNS), the nociceptive-induced activation of the ACC was reduced after treatment. Similar findings were observed in 14 chronic migraine patients treated with eTNS for three months [69]. Comparing 18-fluorodeoxyglucose (^18^FDG) uptake to controls, migraine patients initially displayed hypometabolism of the orbitofrontal cortex and ACC, which reverted after treatment. The ACC is known to be involved in the descending antinociceptive pathway and the orbitofrontal cortex is implicated in cognitive aspects of pain modulation. However, there was no difference between responders and non-responders, possibly due to a small sample size.

Using a single-blind, crossover fMRI study design, Luo and colleagues [70] showed that, compared to sham stimulation, eight minutes of verum electrical stimulation of the auricular branch of the vagus nerve (aVNS) reduced the FC between the amygdala and fronto-parietal brain areas largely involved in pain processing and modulation in 27 migraine patients. Central effects of aVNS have also been investigated in a larger study [71] showing that the increased activity of the thalamus, frontal and parietal areas experienced by 60 migraine patients compared to controls could be reversed after 4 weeks of aVNS treatment. It has also been demonstrated that abnormal activity of the trigeminal cervical complex, insula, cingulate cortex, frontal and temporal gyrus could predict patients´ treatment response to 4-week treatment with aVNS [72].

The study conducted by Markin et al*.* [73] showed that the application of repetitive Transcranial Magnetic Stimulation (rTMS) for five days in 19 migraine patients was associated with FC changes within the default mode, salience and visual networks, which have been implicated in migraine pathophysiology.

Only one study [74] has explored structural brain changes after transcranial direct current stimulation (tDCS), examining 24 patients with migraine who received tDCS or sham stimulation over the visual cortex for 28 days. Compared to sham stimulation, in patients treated with tDCS the frequency of monthly migraine days progressively decreased during the four months after treatment initiation and returned to baseline during the fifth month [75]. Before starting tDCS treatment, migraine patients had decreased GM volume of the left lingual gyrus compared to controls. Five months after treatment, the GM volume of the left lingual gyrus was normalized only in patients who received tDCS but not in those treated with sham treatment. Given the clinical worsening observed after five months from treatment start, these morphometric results are difficult to reconcile with a treatment effect.

The only invasive neurostimulation approach that has been studied using imaging techniques is the occipital nerve stimulation (ONS). Using PET, Matharu and colleagues described an association between pain relief after ONS and regional CBF changes at level of the dorsal rostral pons, ACC, basal ganglia, cuneus, precuneus, cerebellum, frontal, temporal and occipital cortex in a small group of patients with chronic migraine [76].

##### Behavioural approaches

The study of 19 adolescents with migraine using fMRI showed a greater activation of frontal brain regions and an increased FC of the amygdala with frontal and sensorimotor regions after 8-week treatment with cognitive behavioural therapy (CBT), which was significantly associated with headache improvement in terms of reduction of headache days [77]. The amygdala FC with frontal and sensorimotor regions at baseline could predict headache days reduction after treatment [78].

Functional changes in brain areas implicated in the emotional and cognitive aspects of pain have also been demonstrated in 11 adult migraine patients treated for 16 weeks with autogenic training, a behavioural approach that includes desensitization-relaxation techniques [79].

Among CBT strategies, enhanced mindfulness-based stress reduction (MBSR) is an approach based on mindfulness practice and self-compassion that trains the ability to respond to distress [80]. Seminowicz and colleagues described distinct patterns of brain activation during a challenging cognitive task and of RS FC of the insula in 50 migraine patients treated with MBSR compared to 48 patients receiving didactic sessions focused on the role of stress and other triggers in headaches, supporting increased cognitive efficiency after MBSR [81].

### Cluster headache

Using H_2_^15^O-PET, May and colleagues reported that 60 s of hypothalamic deep brain stimulation was able to change the activity of the hypothalamus, thalamus, trigeminal nucleus and ganglion, and several cortical areas that are usually active in pain perception and during CH attacks [40]. However, the study examined only 10 patients and results were uncorrected for multiple comparisons.

Another PET study [41] examined cerebral glucose metabolism in 10 patients with drug-resistant and side-locked chronic CH treated with ONS. Before treatment initiation, CH patients had altered glucose metabolism in pain processing cortical and brainstem areas compared to healthy controls, which normalized after at least 6 months of treatment. No short-term changes were observed, suggesting that ONS may work through slow neuromodulatory processes in CH.

Using ASL, Medina and colleagues [42] examined regional CBF changes before and after greater occipital nerve blockade with methylprednisolone 80 mg and 2 ml of lidocaine 2% in 17 interictal chronic CH patients. Seven days after the blockade, the regional CBF increased in the right secondary visual cortices and decreased in the left medial temporal gyrus, cerebellum, caudate and putamen. At baseline, responders had greater CBF in the right lateral occipital cortex and left medial prefrontal cortex, and lower CBF in the right PCC compared to non-responders. The study underlines that a strictly peripheral treatment can induce measurable central changes.

A recent study [43] examined clinical and brain morphometric predictors of verapamil response, in 194 patients treated for at least three months. Compared to responders, non-responders had an increased GM volume of the cerebellar vermis and of bilateral cerebellar lobule VI, when using an uncorrected threshold. The study showed also that a supervised machine learning algorithm can discriminate verapamil responders from non-responders with an accuracy of 66%, based only on the clinical characteristics of the patients. The inclusion of the cerebellar GM volume in the predictive model increased slightly the accuracy of the verapamil responsiveness prediction (from 66 to 68%). Both accuracies are considered “poor” (< 0.7) according to general guidelines [82].

### Medication overuse headache

Comparing 16 patients with MOH to 68 healthy controls using [26] FDG PET-MRI, Fumal and colleagues [44] found reduced glucose metabolism in pain-related brain areas of patients, including the cerebellum, right parietal cortex, bilateral insula, orbitofrontal cortex, and thalamus. Except for the orbitofrontal cortex, the hypometabolism was reverted after withdrawal. However, the study did not report any restrictions on analgesic intake prior to the scan. It cannot therefore be possible to exclude that metabolic changes observed could be attributed to direct analgesic effects rather than to the overuse of the medication [83].

Similar findings were observed in a later study [45] that examined the BOLD response to a decision making task in patients with MOH who discontinued or not the overused medication. This study found that, compared to controls and chronic migraine patients without MOH, patients with MOH had an increased activity of the ventral medial prefrontal and PCC, which reverted following the medication withdrawal. While, a decreased activity of the midbrain, including the substantia nigra and ventral tegmental area, was specific for MOH and did not change after withdrawal.

Mehnert and colleagues [46] examined changes in GM volume and fMRI response to noxious trigeminal stimuli in 18 patients with MOH before and after withdrawal. After withdrawal, patients displayed an increased responsiveness to nociceptive stimuli and a decreased volume of the left cuneus, superior temporal gyrus, and cerebellum. However, these results should be interpreted with caution given the analgesic intake prior to the pre-withdrawal and follow-up scans and the lack of significant differences in longitudinal fMRI and volumetric changes between patients and controls.

In the study performed by Ferraro et al*.* [47] nine patients with MOH had a higher BOLD response to painful stimulation of the left hand in the primary somatosensory, parietal, and supramarginal cortex, compared with healthy controls. At rescan 3 weeks after withdrawal, this difference disappeared. While this could suggest that withdrawal ameliorated the central sensitization, the relevance of this changed response to extra-cephalic pain in MOH is unknown. However, it is possible that similar differences might occur for cephalic pain.

A few studies have also examined structural brain predictors of withdrawal effect. After medication withdrawal, Riederer et al*.* [48] reported a reduction in midbrain PAG volume specifically in 11 responders. Both Mehnert [46] and Rieder [48] found that a greater volume of the orbitofrontal cortex predicted a better response to withdrawal.

## Discussion

The reviewed studies applied many different imaging approaches, treatment schemes and study designs, leading to results that are often incomparable or inconsistent. Despite this, some coherent findings have been reported for triptans as abortive treatments for migraine attacks, non-pharmacological approaches employed in migraine and cluster headache prevention and for central effects of medication withdrawal in patients with MOH.

In the following, we will discuss evidence coming from the included studies highlighting their strength and weakness.

### Migraine

#### Acute treatments

NSAIDs are the first line acute treatment for migraine [84]. They are thought to act both peripherally and centrally through effects on nociceptive pathways [85]. Their site of action in migraine is unknown, but three studies [16–18] have examined imaging predictors of their efficacy. The findings of these studies may suggest that differences in the FC of the visual network and left amygdala could have importance for the effects of NSAIDs in migraine. While the right amygdala has been implicated in pain-processing, the role of the left amygdala is less clear [86]. Of note, results concerning the visual network were reported at an uncorrected statistical threshold [16] and the inclusion of RS FC metrics of the left amygdala that have already been shown to differ between responders and non-responders might have skewed prediction models [17]. Moreover, the direction of the amygdala RS FC alterations found in patients who responded to NSAID is difficult to interpret since different brain areas were found when comparing healthy controls to the two subgroups of patients.

Ahmed and colleagues [18] showed an association between a poor response to ibuprofen and the presence of WMHs in migraine patients. However, the percentage of consistent responders reported in the study was remarkably high (48.8%) considering that pain freedom at two hours is 20–26% for ibuprofen 200–400 mg [87]. In addition, the number of WMHs increased with age, and age might also affect the efficacy. The findings should be confirmed in a separate cohort and adjusted for age before clinical inferences can be made.

If NSAIDs are inefficient or not tolerated, triptans are the second line acute treatment for migraine [84]. Triptans are 5-HT_1B/1D_ receptor agonists with both vascular and neural effects. An important question is whether different triptans pass the blood–brain barrier (BBB) to exert central effects and side-effects. Imaging studies have provided important information in this regard. The majority of these have used subcutaneous injections of sumatriptan. Using angiography and SPECT, these studies consistently found that sumatriptan constricts extracerebral arteries but do not alter the intracerebral perfusion [19–22]. This suggests that sumatriptan is unable to cross the BBB to an extent where it can act upon the abluminal 5-HT_1B/1D_ receptors of cerebral arteries. Even so, some imaging studies suggest triptans may cross the BBB to some extent and bind centrally, though perhaps not sufficiently to alter the CBF. Two PET studies [23, 25] demonstrated that triptans reduce the rate of cerebral serotonin synthesis and its activity. Deen and colleagues [23] found a 16% reduction of central 5-HT_1B_ receptor binding across pain-modulating brain areas in patients treated with sumatriptan. Serotonin is an inhibitory neurotransmitter, but whether this level of binding is sufficient to inhibit nociceptive signaling is unknown. Importantly, this study was not placebo controlled, so it cannot be completely excluded that increased serotonin binding is part of the untreated migraine attack or that the reduced binding occurred indirectly.

The degree to which the triptans pass the BBB, likely depends on their individual lipophilicity. Almotriptan is the least lipophilic, eletriptan the most, and sumatriptan is in between [88]. BBB passage could explain some differences in efficacy and tolerability. In comparison, lasmiditan, which is lipophilic and designed as an agonist of central 5-HT_1F_ receptors, is efficacious for the treatment of migraine attacks but may possess more marked central side effects than triptans [89]. However, neuroimaging studies examining the central or neurovascular effects of other triptans different from sumatriptan and eletriptan are lacking, and none have examined those of lasmiditan.

Interestingly, findings with DHE, an effective migraine treatment that also activates 5-HT_1B_ receptors, suggests that high efficacy can be reached through peripheral mechanisms of action alone [26]. Ergotamine use, however, is hampered by potentially serious side effects.

### Preventive treatments

#### Pharmacological approaches

Numerous treatments are approved for migraine prevention. The response to these treatments is generally heterogenous and there are few clinical predictors of treatment response. Preventives may work at different levels of the signaling pathways driving migraine pathogenesis, which may, in part, explain variability between patients [90]. Neuroimaging offers the possibility to identify these sites of action, and how they differ between responders and non-responders.

The findings of the two studies [28, 29] investigating central effects of beta-blockers could suggest that these treatments primarily act peripherally in migraine. However, the studies’ small sample sizes preclude firm conclusions. The beta-blockers discussed are all lipophilic and capable of passing the BBB. Future studies might further explore such direct or indirect central effects of beta-blockers.

Flunarizine is a calcium antagonist which also blocks H1, serotonin, and D2 receptors in addition to voltage gated-sodium channels [91]. Because of the multifarious effects, the exact mechanisms of action in migraine are unknown. However, Wöber et al*.* [31] speculated that the anti-dopaminergic effects could be mainly involved in migraine prevention.

Botulinum toxin is an effective treatment option for chronic migraine. Botox is administered subcutaneously, where it inhibits the release of vasodilatory neurotransmitters involved in migraine [92]. Because of this, its primary site of action is thought to be peripheral, with secondary central effects. Due to the logistical and financial demands of the treatment, predictors of treatment response are highly relevant. Hubbard and colleagues suggested that functional and structural changes in pain and visual processing areas could have a role in determining botox efficacy [33]. However, their study compared only 11 responders to 12 non-responders, which is likely too few for generalizable results.

Several randomized controlled trials (RCT) demonstrated that mAbs targeting the CGRP are effective and well-tolerated migraine preventive treatments [93]. Their site of action is thought to be mainly in the periphery. However, recent fMRI [34–36] and MRS [37] studies demonstrated that anti-CGRP mAbs modulate the activity of pain related brain areas. Central effects may occur secondary to peripheral modulation, or directly through the negligible fraction of mAbs that crosses the BBB [94].

Imaging might help to identify responders to anti-CGRP mAbs. This is highly warranted, since their high-cost hampers widespread use. Distinct patterns of brain functional activity have been found in patients treated with erenumab and galcanezumab. Differences between galcanezumab and erenumab in treatment-related functional brain changes are interesting, since they could explain why some patients have distinct responses to mAbs targeting the CGRP ligand and those blocking the receptor [95]. However, the major limitation of these studies are the small sample size and the use of uncorrected statistical comparisons, which have a high risk of false positive findings [96]. As of date, no studies have reported central changes with anti-CGRP mAbs using an appropriately corrected approach, where false positives can be excluded with greater certainty.

#### Non-pharmacological approaches

The poor compliance of patients to some pharmacological treatments due to adverse effects and contraindications linked to pregnancy or lactation have encouraged the use of non-pharmacological approaches for migraine prevention [97]. Treatments that have been examined using imaging techniques include acupuncture, behavioural and neuromodulation approaches.

Acupuncture involves the stimulation of specific points on the body by the insertion and rotation of filiform needles until a sensation of numbness and distention, called the de-qi sensation, is achieved [98]. Although acupuncture remains one of the most frequently used approaches in Chinese medicine [98], the use of acupuncture in migraine prophylaxis has yielded to contradictory results. A large, multicentre, RCT did not find acupuncture to be superior to sham [99]. This corroborates a Cochrane meta-analysis that identified several differences in methodology and outcome selection [100]. Imaging findings related to acupuncture should therefore be interpreted in light of the uncertain role of acupuncture in migraine prevention.

In recent years, a vast number of neuroimaging studies have explored the neural mechanisms of acupuncture. Some studies [50–57]  suggested that acupuncture could promote migraine improvement by modulating the activity of migraine-affected nociceptive regions and enhancing the function of the descending pain inhibitory system. One of the main limitations of these studies was the lack of a sham group, thus not allowing the exclusion of a placebo effect. However, similar evidence were also found when the effects of verum acupuncture was compared to sham acupuncture. Widespread brain functional and metabolic changes and a reinforced pain inhibitory activity of the brainstem was found in patients treated with verum acupuncture compared to those receiving sham acupuncture. These findings may suggest that only verum acupuncture could modulate the activity of pain-related brain areas, thus improving migraine.

Although further larger RCTs on non-invasive neuromodulation techniques are needed, their potential as therapeutic alternatives to standard pharmacological treatments have recently emerged [101]. Many neuromodulation devices have been introduced in the management of migraine patients. They work by stimulating the central or peripheral nervous system with electric or magnetic stimuli, thus modulating central mechanisms involved in migraine [97].

Transcutaneous cranial nerve stimulation, such as the eTNS and aVNS, modulates the nerves activity at the periphery by applying an electrical current [102]. The aVNS stimulates the auricular branch of the vagus nerve at the concha of the outer ear. This branch of the vagus nerve contains less myelinated Aβ fibers compared to the cervical branch [103, 104]. These anatomical differences may explain the different stimulation regimen used for aVNS and cervical VNS [105]. During aVNS electrical pulses at 25 Hz are applied to the skin of the concha for 1–4 hours [106]. While, cervical VNS stimulation lasts for 2 min, it can be performed 6–12 times a day and delivers a maximum output current of 60 mA to the anterolateral surface of the vagus nerve in the neck [105, 107].

Using fMRI and PET, a few studies [68–71] showed that both eTNS and aVNS could exert their beneficial migraine preventive effect turning the activity of pain modulatory brain areas, including the ACC, thalamus and trigeminal cervical complex, to normal. Even so, this evidence should be confirmed by further larger studies with a sufficient sample of responders and non-responders. rTMS uses a fluctuating magnetic field to produce an electrical current that can change the excitability of brain networks [102]. Another non-invasive neuromodulation method is the tDCS, which modulates the cortical activity by applying an excitatory or inhibitory electric current to the scalp [102]. Only two studies [73, 74] have investigated functional and structural brain changes related to rTMS and tDCS treatments. The small sample size of these studies, the lack of a control group and the use of an uncorrected statistical threshold disallow solid conclusions regarding central modifications related to these treatments.

The ONS involves an implantable device that delivers electrical stimulation to the greater occipital nerve. One PET study [76] including chronic migraine patients with implanted ONS showed that treatment-related pain improvement correlated with CBF changes in regions involved in the affective dimension of pain and migraine pathophysiology. The study's extremely small sample size hinders drawing conclusions that can be applied broadly. Morevoer, its shoud be noted that results from three RCTs examining the efficacy of ONS in migraine prevention have overall not been promising [102].

Behavioural approaches, including relaxation and CBT, have been used in the management of migraine patients with the aim of teaching patients how to cope with the experience of pain and other migraine symptoms [108]. Despite the lack of high quality evidence supporting their effectiveness in migraine prevention, behavioural treatments remain an important choice for many patients [108]. fMRI studies [77–81] showed that behavioural approaches may influence the cognitive and emotional control of pain to aid migraine improvement.

### Cluster headache

Imaging data investigating central effects of CH treatments are scarce and with small samples, thus limiting interpretation. Two PET studies [40, 41] examined neural substrates of neurostimulation in CH, showing treatment-related changes in the activity and metabolism of brain areas implicated in pain transmission and CH attacks. Interestingly, Tso and colleagues [43] showed that clinical characteristics of CH have a rather low accuracy (66%) in discriminating patients who respond to verapamil, a calcium channel blocker that is the first-line preventive drug for CH, from non-responders. The accuracy of the verapamil responsiveness prediction was marginally increased (from 66 to 68%) when clinical features were combined with the cerebellar GM volume. These findings suggest that structural MRI has a minimal role in predicting response to verapamil apart from what can be clinically deduced. However, the study was limited by inclusion of patients with probable and post-traumatic CH, retrospective acquisition of data, and missing information on whether patients were in or out of bout. Furthermore, the study also used different scanners with different field strengths. Though the statistical analysis attempted to adjust for this, no harmonization efforts were reported.

### Medication overuse headache

MOH is a secondary headache disorder attributed to overuse of acute headache treatments in patients with a pre-existing headache disorder. Medication withdrawal is crucial in the management of MOH, since it reverts the condition in most patients. The exact mechanisms underlying MOH are unknown. Possible pathophysiological mechanisms may involve the interaction between central sensitization, altered descending pain modulation, biopsychosocial and genetic factors, that affect a state of vulnerability [109]. Imaging before and after withdrawal is instrumental because it could provide information regarding central mechanisms predisposing to the condition and those that are secondary to the frequent intake of acute treatments.

Findings from MRI and PET studies [44–48] indicate that the abnormal function and metabolism of pain processing regions tend to normalize following the discontinuation of the overused treatment, suggesting that these alterations may be secondary to the frequent intake of acute therapies. Whereas, abnormalities of brain regions implicated also in drug dependence, such as the orbitofrontal cortex and ventral tegmental area, tend to persist despite the medication withdrawal, thus reflecting an underlying liability to medication overuse. Curiously, all studies [44, 46, 48] investigating MOH susceptibility and predictors of withdrawal effect implicated the orbitofrontal gyrus, possibly reflecting that the more susceptible patients are also less effective at withdrawing.

## Conclusions

In recent years, an increasing number of imaging studies have sought to clarify central mechanisms of action of pharmacological and nonpharmacological treatments commonly used to treat headache patients. It is not unexpected that most of the studies were focused on migraine, being the most frequently studied form of headache. However, if we look at the individual type of acute and preventive migraine treatment, there are only a few studies available, except for acupuncture.

The results of this systematic review suggest that triptans may cross the BBB to some extent, though perhaps not sufficiently to alter the intracranial CBF. An interesting goal of future imaging studies would be to examine how triptans with different efficacy and tolerability cross the BBB. Furthermore, central and vascular mechanisms of action of novel migraine abortive medications, the gepants and lasmiditan, remain unexamined.

In migraine prevention, there is a great need for imaging studies on established treatment, such as anti-hypertensives and anti-epileptics, to further our understanding of their mechanism of action. Whereas imaging studies have provided important information about the anti-CGRP monoclonal antibodies, large-scale studies with robust statistical inferences are needed to consolidate and verify prior findings. This may, in the future, facilitate development of clinically useful predictors of efficacy that can personalize treatment of headache patients.

Acupuncture in migraine, neuromodulation in migraine and cluster headache patients, and medication withdrawal in patients with MOH could lead to headache improvement by reverting headache-affected pain processing brain areas. The way in which neuromodulation devices acting at the periphery could exert their central effects need to be clarified. Moreover, future studies should explore the potential effects of combined pharmacological and non-pharmacological approaches on the brain. Yet, there are no clearly defined brain regions in which each treatment acts, and there are no imaging patterns that could firmly predict the effectiveness of medications.

It should be noted that the studies included in the present review were extremely heterogeneous regarding treatment schemes, study designs, included subjects, and imaging techniques employed. Other limitations of the currently available literature are the small sample size and the frequent use of inadequate statistical approaches that introduce a considerable risk of false positive findings. For many treatment approaches, this excludes robust conclusions. Future studies with adequate sample size, reproducible study paradigms and homogeneous study populations are needed. Moreover, in the future more efforts should be made to study patients with trigeminal autonomic cephalalgias or post-traumatic headache.

A better understanding of how headache treatments work along with the identification of biomarkers of patients’ response could yield crucial insights into the biological mechanisms underlying the pathophysiology of headaches.

## Supplementary Information

**Additional file 1:** **Supplementary Table 1.** Search string used for PubMed and Embase databases. **Supplementary Table 2.** Acupuncture for migraine prophylaxis. **Supplementary Table 3.** Non-invasive and invasive neuromodulation techniques for migraine prophylaxis. **Supplementary Table 4.** Behavioral approaches for migraine prophylaxis.

## Data Availability

Data are available from the corresponding author upon reasonable request.

## Abbreviations

ACC: Anterior cingulate cortex
ASL: Arterial spin labeling
aVNS: Auricular vagus nerve stimulation
BBB: Blood–brain barrier
BOLD: Blood oxygenation level dependent
CBF: Cerebral blood flow
CBT: Cognitive behavioural therapy
CGRP: Calcitonin gene-related peptide
CH: Cluster headache
Cho: Choline
Cr: Creatine
DHE: Dihydroergotamine
eTNS: External trigeminal nerve stimulation
FC: Functional connectivity
^18^FDG: 18-Fluorodeoxyglucose
fMRI: Functional magnetic resonance imaging
GABA: Gamma-aminobutyric acid
Glx: Glutamate-glutamine
GM: Grey matter
GTN: Nitroglycerin
mAbs: Monoclonal antibodies
MBSR: Mindfulness-based stress reduction
MOH: Medication overuse headache
MRI: Magnetic resonance imaging
MRS: Magnetic resonance spectroscopy
NAA: N-acetylaspartate
NSAIDs: Non-steroidal anti-inflammatory drugs
ONS: Occipital nerve stimulation
PAG: Periaqueductal grey
PCC: Posterior cingulate cortex
PET: Positron emission tomography
RCT: Randomized controlled trial
RS: Resting state
rTMS: Repetitive transcranial magnetic stimulation
SBM: Surface-based morphometry
SPECT: Single-photon emission computerized tomography
tDCS: Transcranial direct current stimulation
USPIO: Ultrasmall superparamagnetic iron oxide
VBM: Voxel-based morphometry
WMHs: White matter hyperintensities
