# Supplementary material for: Imaging the brain and vascular reactions to headache treatments: a systematic review
Source: J Headache Pain. 2023 May 24;24(1):58. doi: 10.1186/s10194-023-01590-5 (PMC10207747; doi:10.1186/s10194-023-01590-5)
Supplement: Supplementary file 1 — Additional file 1: Supplementary Table 1. Search string used for PubMed and Embase databases. Supplementary Table 2. Acupuncture for migraine prophylaxis. Supplementary Table 3. Non-invasive and invasive neuromodulation techniques for migraine prophylaxis. Supplementary Table 4. Behavioral approaches for migraine prophylaxis. [file 10194_2023_1590_MOESM1_ESM.docx]

**Supplementary Table 1. Search string used for PubMed and Embase databases.**

| Search | Database | Query | Results (number) |
| --- | --- | --- | --- |
| #1 | PubMed | Search: (magnetic resonance imaging OR positron emission tomography OR single-photon emission computerized tomography OR MRI OR PET OR SPECT) AND (Migraine OR tension type headache OR cluster headache OR hemicrania continua OR paroxysmal hemicrania OR medication overuse OR posttraumatic headache) AND (acute therapy OR acute treatment OR preventive therapy OR preventive treatment OR non-pharmacological OR triptan OR NSAIDs OR non-steroidal anti-inflammatory drugs OR steroid OR indomethacin OR gepants OR lasmiditan OR antiepileptics OR calcium channel blocker OR antidepressants OR beta blockers OR anti-hypertensive OR monoclonal antibodies OR oxygen OR anti serotoninergic OR acupuncture OR neurostimulation OR cognitive behavior therapy). Sort by: Most Recent | 948 |
| #2 | Embase | Search: (magnetic resonance imaging OR positron emission tomography OR single-photon emission computerized tomography OR MRI OR PET OR SPECT) AND (Migraine OR tension type headache OR cluster headache OR hemicrania continua OR paroxysmal hemicrania OR medication overuse OR posttraumatic headache) AND (acute therapy OR acute treatment OR preventive therapy OR preventive treatment OR non-pharmacological OR triptan OR NSAIDs OR non-steroidal anti-inflammatory drugs OR steroid OR indomethacin OR gepants OR lasmiditan OR antiepileptics OR calcium channel blocker OR antidepressants OR beta blockers OR anti-hypertensive OR monoclonal antibodies OR oxygen OR anti serotoninergic OR acupuncture OR neurostimulation OR cognitive behavior therapy). | 1477 |

**Abbreviations:** MRI **=** Magnetic resonance imaging; NSAIDs = Non-steroidal anti-inflammatory drugs; PET = Positron emission tomography; SPECT = Single-photon emission computerized tomography

**Supplementary Table 2. Acupuncture for migraine prophylaxis.**

| **Reference** | **Population** | **Treatment** | **Imaging** | **Results** | **Limitations** |
| --- | --- | --- | --- | --- | --- |
| Tian *et al.*, 2021^51^ | 48 MO (19 responders, 29 non-responders)  60 HC | **Type:** Acupuncture  **Duration**: 4 weeks: 20 sessions lasting 30 minutes per week  **Response definition:** 30% reduction in headache intensity or headache frequency | **Modality**: RS fMRI  **Time-points:** Before and after treatment for patients  HC underwent one scan | **Baseline: Patients vs HC**  ↑ FC between cingulate and superior frontal gyri compared to HC  ↓ FC of the cingulate gyrus with the insula and inferior parietal lobe, as well as between the middle frontal gyrus and inferior parietal lobe compared to HC  **Baseline-to-follow-up:**  ↑ FC between amygdala and middle frontal gyrus, hippocampus and insula, inferior parietal lobe and insula, middle frontal gyrus and thalamus in patients after treatment  ↓ FC of the amygdala with the insula and superior frontal gyrus, as well as between the cingulate gyrus and superior frontal gyrus, hippocampus, and thalamus in patients after treatment  **Baseline-to-follow-up: Responders *vs* non-responder**  ↑ FC between distinct thalamic subregions in responders | No sham acupuncture group |
| Zhang *et al.*, 2016^52^ | 12 MO  12 HC | **Type:** Acupuncture  **Duration**: 4 weeks: 5 sessions lasting 30 minutes per week  **Response definition:**  Pain intensity, duration, and frequency of migraine attacks | **Modality**: RS fMRI  **Time-points:** Before and after treatment for patients  HC underwent one scan | **Baseline: Patients vs HC**  ↓ Activation in the bilateral frontal gyrus, parietal lobule, cingulate cortex, precuneus, supramarginal and temporal gyrus in patients compared to HC  **Baseline-to-follow-up:**  ↑ Activation in the bilateral superior frontal gyrus, medial frontal gyrus, precuneus, inferior parietal lobule, cingulate gyrus, superior temporal gyrus, middle temporal gyrus, and supramarginal gyrus in patients after treatment | No sham acupuncture group  Small sample size  No correlation with clinical features |
| Chen *et al.*, 2022^53^ | 40 MO  36 HC | **Type:** Acupuncture  **Duration**: 5 weeks: 12 sessions, twice a week, lasting 20 min  **Response definition:** frequency of migraine, pain severity based on VAS,  self-assessing questionnaires for depression and anxiety (SAS and SDS) and patients’ quality of life (MSQ) | **Modality**: RS fMRI  **Time-points:** Before and after the first and the last acupuncture treatment for MO  HC underwent one scan | **Baseline: Patients vs HC**  ↑ Activation in the left inferior occipital gyrus in patients compared to HC  ↓ Activation in the RVM, left cerebellum, right inferior frontal gyrus, right cingulate, right precuneus, left inferior parietal, supramarginal and angular gyri in patients compared to HC  **Baseline-to-follow-up:**  ↑ Activation in the RVM, superior lobe of the left cerebellum and right precuneus in patients after treatment  ↑ FC from the RVM to the to the right middle frontal gyrus, left insula, right precentral gyrus, and right supramarginal gyrus after treatment  ↑ FC from the left Cerebellum to the left middle occipital gyrus and for the right precuneus to the right thalamus after treatment  **Clinical correlation**  The increased FC of the RVM was positively correlated with MSQ scores and negatively correlated with migraine attack frequency and VAS scores | No sham acupuncture group |
| Li *et al.,* 2016^54^ | 100 MO interictal (VA n=35, SA n=11, No Treatment (NT) n=16)  42 HC | **Type:** Verum (VA) and sham acupuncture (SA)  **Duration**: 4 weeks: 20 sessions lasting 30 min (once per day for five days followed by a two-day break)  **Response definition:** pain severity based on VAS,  self-assessing questionnaires for depression and anxiety (SAS and SDS), headache frequency | **Modality**: RS fMRI  **Time-points:** Before and after treatment for patients  HC underwent one scan | **Baseline: Patients vs HC**  ↑ FC between the ventrolateral PAG and the bilateral adjacent PAG in patients compared to HC  ↓ FC between the ventrolateral PAG and the bilateral medial prefrontal cortex, left orbitofrontal cortex and rostral anterior cingulate cortex in patients compared to HC  **Baseline-to-follow-up: VA and SA**  ↑ FC between ventrolateral PAG and the bilateral middle cingulate cortex and rostral anterior cingulate cortex and left medial prefrontal cortex after treatment in the VA and SA group  **Baseline-to-follow-up: VA and SA vs NT**  ↑ FC between ventrolateral PAG and the bilateral rostral anterior cingulate cortex, left medial prefrontal cortex and left middle cingulate cortex after treatment in patients receiving the VA compared to those patients who did not receive any treatment  **Clinical correlation:**  Negative correlation between FC changes observed after treatment in VA and SA group and changes in headache intensity after treatment | No comparison between VA and SA group |
| Zou *et al.*, 2019^55^ | 14 CM  18 HC | **Type:** Acupuncture  **Duration**: 3 months: 36 sessions, lasting 30 minutes, three times per week  **Response definition:** pain severity based on VAS, monthly headache attacks, monthly headache days, monthly amount of acute headache medications | **Modality**: RS fMRI  **Time-points:** Before and after treatment for patients  HC underwent one scan | **Baseline: Patients vs HC**  ↓ Activation of the left superior frontal gyrus and left precuneus in the DMN in patients compared to HC  **Baseline-to-follow-up:**  ↑ FC of the left superior prefrontal gyrus and left precuneus within the DMN in patients after treatment | No sham acupuncture group  Small sample size |
| Li *et al.*, 2015^56^ | 12 MO  12 HC | **Type:** Acupuncture  **Duration**: 4 weeks: 5 sessions lasting 30 min per week | **Modality**: RS fMRI  **Time-points:** Before and after treatment for patients  HC underwent one scan | **Baseline: Patients vs HC**  ↓ FC between the right FPN and the left precentral gyrus, supramarginal gyrus, inferior parietal lobule, and postcentral gyrus in patients compared to HC  **Baseline-to-follow-up:**  ↑ FC between the right FPN and the left precentral gyrus, inferior parietal lobule and postcentral gyrus after treatment | No sham acupuncture group  Small sample size  No clinical correlation with response to treatment |
| Ishiyama *et al.*, 2022^57^ | 20 Mx (3 MA, 11 MO and 6 CM)  23 HC | **Type:** C2 peripheral nerve field stimulation with electroacupuncture  **Duration**: 3 months, electroacupuncture was performed once a week **Response definition:** headache intensity based on numeric rating scale (NRS), headache impact (HIT-6 score), self-assessing questionnaires for depression (SDS) | **Modality**: RS fMRI  **Time-points:** Before and after treatment for patients | **Baseline: CM vs HC and vs MO/MA**  ↑ FC of the left amygdala with the bilateral thalamus and posterior cingulate cortex in CM patients compared to HC  ↓ FC of the right hypothalamus with bilateral post-central gyrus and right amygdala in CM patients compared to HC  ↓ FC of the right hypothalamus with the right insula, bilateral postcentral gyrus, and amygdala in CM patients compared to MO/MA  **Baseline-to-follow-up:**  In MO patients, ↓ FC of the right hypothalamus with the left insula after treatment  No significant changes in CM after treatment | No sham acupuncture group  Small sample size |
| Li *et al.*, 2017^58^ | 100 MO (VA n=60, SA n=20, No Treatment (NT) n=20)  46 HC | **Type:** Verum (VA) and sham acupuncture (SA)  **Duration**: 4 weeks: 20 sessions of acupuncture lasting 30 min  **Response definition:** pain severity based on VAS,  self-assessing questionnaires for depression and anxiety (SAS and SDS), headache frequency | **Modality**: RS fMRI  **Time-points:** Before and after treatment for patients  HC underwent one scan | **Baseline: Patients vs HC**  ↓ FC between the right FPN and the precuneus, lingual and fusiform gyrus, cuneus, temporal areas, secondary somatosensory cortex, cerebellum, and inferior occipital gyrus in patients compared to HC  **Baseline-to-follow-up: VA and SA**  In the VA and SA group, ↓ FC between the right FPN and bilateral precuneus, right paracentral gyrus and postcentral gyrus  **Clinical correlation:**  Positive correlation between FC changes observed after treatment in VA and SA group and changes in headache intensity after treatment  **Baseline-to-follow-up:** **VA and SA vs NT**  ↑ FC in the left cerebellum and right middle frontal gyrus in patients treated with VA and SA, compared to those who did not receive any treatment |  |
| Liu *et al.*, 2021^59^ | 40 MO  16 HC | **Type:** Acupuncture  **Duration**: 6 weeks: 12 sessions 2 days per week,  followed by 24 weeks of follow-up  **Response definition: N**umber of migraine days and average of severity of headache | **Modality**: RS fMRI  **Time-points:** Before and after treatment for patients  HC underwent one scan | **Baseline: Patients vs HC**  ↓ Activation of the cerebellum in patients compared to HC  **Baseline-to-follow-up:**  ↑ Activation of the cerebellum and angular gyrus in patients after treatment  **Clinical correlation**  Changes in the functional activity of the angular gyrus were positively correlated with migraine days at baseline and with changes in migraine attack frequency after treatment | No sham acupuncture group |
| Gu *et al.*, 2018^60^ | 45 patients (15 MO, 15 cervicogenic headache (CH))  15 HC | **Type:** Verum acupuncture for patient group, sham acupuncture in HC  **Duration**: 9 weeks: 5 sessions per week | **Modality**: ^1^H-MRS for NAA, Cr and Cho  **Time-points:** Before and after treatment for patients | **Baseline-to-follow-up:**  ↑ Levels of NAA/Cr in the thalamus in MO patients after treatment  ↓ Levels of NAA/Cr in the anterior cingulate cortex in the CH group and ↑ levels of NAA/Cr of the anterior cingulate cortex in HC after treatment  No changes in NAA/Cr levels of the anterior cingulate cortex in migraine patients  No changes in NAA/Cr levels of paracentral gyrus and no changes in Cho/Cr levels in any groups  **Clinical correlation**  In all patients, thalamic NAA/Cr levels correlated with baseline and after-treatment pain severity | No sham group in patients  Longitudinal changes were assessed only within group but not between-group |
| Zhang *et al.*, 2021^61^ | 44 menstrual MO | **Type:**  24 verum (VA) and 20 Sham Acupuncture (SA)  **Duration**: 3 months: interventions were performed 1 week before menses, once every other day  **Response definition:**  Frequency of migraine attacks, pain severity based on VAS,  self-assessing questionnaires for depression and anxiety (SAS and SDS), | **Modality**: RS fMRI  **Time-points:** Before and after treatment for patients | **Baseline:**  The activity of the right precuneus was positively correlated with the presence of depression in patients  The activity of the right middle temporal gyrus was negatively correlated with pain severity in patients  **Baseline-to-follow-up: VA vs SA**  ↑ Activation of the right middle frontal gyrus in patients treated with VA  ↓ Activation of the left anterior cingulate cortex and right inferior frontal gyrus in patients treated with VA  ↑ Activation of the right superior frontal gyrus, left cuneus, and right middle frontal gyrus in patients treated with VA  ↓ Activation of the right superior temporal gyrus in patients treated with VA  **Clinical correlation**:  The altered activity of the left anterior cingulate cortex was positively correlated with the anxiety and depression improvement in the VA group | No HC group  Differences in hormone levels between VA and SA groups were not assessed |
| Zhao *et al.*, 2014^62^ | 80 MO, but MRI obtained only from 40 MO | **Type:** 20 Verum acupuncture (VA) and 20 sham acupuncture (SA)  **Duration**: 8 weeks: 32 sessions lasting 30 min, 4 sessions per week  **Response definition:** intensity of pain (VAS score), frequency of migraine attacks, headache impact quantified using Headache Impact Test-6 (HIT-6) questionnaire | **Modality**: RS fMRI  **Time-points:** Before and after treatment for patients | **Baseline-to-follow-up: VA vs SA:**  ↑ Activation of the bilateral anterior cingulate cortex, insula, thalamus, superior temporal gyrus, cuneus, lingual gyrus, cerebellum and brainstem after verum treatment  ↓ Activation of the bilateral posterior cingulate cortex, middle frontal gyrus, angular gyrus, precuneus, middle temporal gyrus, left hippocampus, inferior parietal lobule, inferior temporal gyrus and right postcentral gyrus after verum treatment | No HC group |
| Liu *et al.*, 2022^63^ | 40 MO | **Type:** 20 verum (VA) and 20 sham acupuncture (SA)  **Duration**: 4 weeks: 12 sessions of 30 min  **Response definition** monthly migraine days, pain severity (VAS score), headache impact (HIT-6), patients’ quality of life (MSQ), Beck depression Scale-II (BDI-II) and Beck anxiety scale (BAI), Pittsburgh sleep quality Index (PSQI), Montreal cognitive assessment (MoCA) | **Modality**: RS fMRI  **Time-points:** Before and after treatment for patients | **Baseline-to-follow-up: VA vs SA**  ↑ FC between the amygdala and the left middle temporal gyrus after VA treatment  ↑ FC between the middle cingulate cortex and the insula, bilateral precentral gyrus, bilateral middle cingulate cortex, and right postcentral gyrus after VA treatment  ↑ FC between prefrontal cortex and the cerebellum, bilateral medial superior frontal gyrus, bilateral middle frontal gyrus, right middle temporal gyrus, right middle occipital gyrus, right precuneus and left angular after VA treatment  **Clinical correlation**  Negative correlation between clinical effects and increased FC between the right amygdala/cingulate cortex and the left middle temporal gyrus  **Prediction of treatment response**  Baseline FC between the right amygdala and left middle temporal gyrus could predict treatment response | No HC group  Short-term effect of acupuncture  Migraine phase (ictal or interictal) at the time of the fMRI was not considered |
| Li *et al.*, 2017^66^ | 62 MO (VA n=35, SA n=11, No Treatment (NT) n=16)  42 HC | **Type:** Verum acupuncture (VA) and sham (SA)  **Duration**: 4 weeks: 20 sessions of 30 min, 1 session per week  **Response definition:** Migraine intensity and frequency | **Modality**: RS fMRI  **Time-points:** Before and after treatment for patients  HC underwent one scan | **Baseline: Patients vs HC**  ↑ Activation of the left posterior insula and left putamen/caudate in patients compared to HC  ↓ Activation of the bilateral middle occipital cortex/cuneus and bilateral RVM/TCC in patients compared to HC  **Baseline-to-follow-up: VA and SA**  In the VA group, ↑ activation in the bilateral RVM/TCC after VA treatment  In the SA group, ↓ FC in the bilateral RVM/TCC after VA treatment | Small sample size for subgroups of patients  No evaluation of de-qi sensation |
| Yang *et al.*, 2012^65^ | 30 MO (10 No Treatment (NT), 10 VA, 10 SA) | **Type:** Electroacupuncture performed on:  -Traditional Acupuncture Group (verum acupuncture VA),  -Control Acupuncture Group (sham, SA) (non-specific acupoints)  **Duration**: 30 min  **Response definition:** VAS intensity pain | **Modality**: ^18^FDG-PET/CT  **Time-points:** During acupuncture simulation and lasting 30 min | **VA vs NT groups:**  ↑ Metabolism in the middle temporal cortex, orbital frontal cortex, insula, middle frontal gyrus, angular gyrus, posterior cingulate cortex, precuneus and middle cingulate cortex in VA group compared to NT group  ↓ Metabolism in the parahippocampus, hippocampus, fusiform gyrus, postcentral gyrus, and cerebellum in VA group compared to NT group  **SA vs NT:**  ↑ Metabolism in the middle temporal cortex, supratemporal gyrus, supramarginal gyrus and middle cingulate cortex after SA treatment  ↓ Metabolism in the cerebellum after SA treatment | No HC group  Small sample size for the subgroups of patients  No clinical correlation |
| Yang *et al.*, 2014^64^ | 30 MO (10 No Treatment (NT), 10 VA, 10 SA | **Type:** Verum electro-acupuncture (VA) and sham acupuncture (SA)  **Duration**: 30 min  **Response definition:** Pain intensity (VAS score) | **Modality**: ^18^FDG-PET/CT  **Time-points:** During acupuncture simulation and lasting 30 min | **VA vs NT group:**  ↑ Metabolism in the middle frontal gyrus, postcentral gyrus, the precuneus, parahippocampus, cerebellum and middle cingulate cortex in patients treated with VA compared to NT group  ↓ Metabolism in the left hemisphere of middle temporal cortex of patients treated with VA compared to NT group  **SA vs NT group:**  ↑Metabolism in the poster cingulate cortex, insula, temporal gyrus, postcentral gyrus, fusiform gyrus, parietal lobe, supramarginal gyrus, middle occipital lobe, angular and precuneus in patients treated with SA compared the NT group  ↓ Metabolism in the cerebellum and parahippocampus in patients treated with SA compared the NT group | No HC group  No clinical correlation  Small sample size for the subgroups of patients |
| Yang *et al.*, 2020^68^ | 80 MO | **Type:** 41 verum acupuncture (VA) and 39 sham (SA)  **Duration**: 4 weeks: 3 sessions lasting 30 min  **Response definition:** 50% reduction of migraine days | **Modality**: T1w MRI  **Time-points:** Before and after treatment for patients | **Prediction of treatment response: VA group**  In the VA group, a predictive model including GM volume of the calcarine cortex, precuneus, cuneus, temporal, frontal and parietal gyrus could discriminate responders from non-responders with and accuracy of 83%  Baseline GM volume of all predictive regions was significantly different between responders and non-responders  Compared to non-responders, patients in the responder group experienced increased GM volume of the left cuneus after treatment  **Clinical correlation: VA group**  Baseline GM volume of the cuneus, frontal and parietal gyrus was correlated with reduction in migraine days after treatment | No HC group  A validation cohort is missing |

Abbreviations: BAI= Beck anxiety scale; BDI-II= Beck depression Scale-II; CH = cervicogenic headache; Cho = choline; Cr = Creatinine; CM = Chronic migraine; DMN= Default mode network; FC= Functional connectivity; ^18^FDG = 18-fluorodeoxyglucose; fMRI = functional magnetic resonance imaging; GM= Grey matter; HIT-6= Headache Impact Test-6; NAA = N-acetyl aspartate MA= migraine with aura; MO= migraine without aura; MoCA= Montreal cognitive assessment; MRI= magnetic resonance imaging; Mx= migraine with or without aura; MSQ= Migraine-Specific Quality of Life Questionnaire; PAG= Periaqueductal gray; PSQI= Pittsburgh sleep quality Index; FPN= Frontoparietal network; PET= positron emission tomography; RS= resting-state; RVM= Rostral ventromedial medulla; SAS= Self-Rating Anxiety Scale; SDS= Self-Rating Depression Scale; SPECT= single-photon emission computed tomography; T1w= T1-weighted; TCC = Trigeminocervical complex; VA = Verum acupuncture; VAS= Visual Analog Scale; SA = Sham acupuncture.

**Supplementary Table 3. Non-invasive and invasive neuromodulation techniques for migraine prophylaxis.**

| **Reference** | **Population** | **Treatment** | **Imaging modality** | **Results** | **Limitations** |
| --- | --- | --- | --- | --- | --- |
| Russo *et al.*, 2017^69^ | 16 MO interictal  16 HC | **Type:** Electrical external trigeminal neurostimulation (eTNS) using Cefaly^©^ device  **Duration:** 20 min application per day for 2 months  **Response definition:** 50% reduction of monthly migraine attacks and migraine days | **Modality**: fMRI during trigeminal heat stimulation  **Time-points**: Before and after treatment MRI in patients  HC underwent only one MRI | **Baseline: Patients vs HC**:  ↑ Activation of the perigenual part of the right ACC in migraine patients compared to controls  **Baseline-to-follow-up:**  ↓ Activation of the perigenual part of the right ACC in migraine patients after treatment  **Clinical correlation:**  Association between ACC activity and migraine attack frequency at baseline  Association between ACC activity and migraine attack frequency after treatments | No sham device  HC did not undergo eTNS treatment |
| Magis *et al.*, 2017^70^ | 14 MO  20 HC | **Type**: Electrical external Trigeminal Nerve Stimulation (eTNS) using Cefaly^©^ device  **Duration**: 20 min application per day for 3 months  **Response definition:** 50% reduction of monthly migraine  attacks | **Modality:** ^18^FDG-PET  **Time-points:** MO underwent three PET scans: at baseline, immediately after a 1-hour session of eTNS and after three months of daily eTNS therapy  HC had only one PET scan | **Baseline: Patients vs HC**  ↓ Metabolism in the orbitofrontal cortex and rostral ACC in patients compared to HC  **Baseline-to-follow-up:**  ↑ Metabolism in the orbitofrontal cortex and rostral ACC after treatment | No sham device  Small sample size |
| Luo *et al.*, 2020^71^ | 27 MO  (interictal) | **Type:** Real transcutaneous auricular vagus nerve stimulation (aVNS) applied at the left cymba concha (with vagal fibers) and sham-aVNS applied on left scapha (no vagal afferent fibers)  **Duration:** 8 min continuous stimulation | **Modality:** RS fMRI  **Time-points:** During verum and sham aVNS | **Treatment vs sham:**  ↓ FC between the left amygdala and the left frontal gyrus, right supplementary motor area and bilateral paracentral lobule after treatment compared to sham  ↓ FC between the right amygdala and with the left middle frontal gyrus after treatment compared to sham | No HC group  No Clinical response assessment |
| Feng *et al.*, 2022^72^ | 60 MO (interictal)  60 HC | **Type**: Auricular vagus nerve electrical stimulation at the left cymba concha  **Duration**: 4 weeks: 12 treatment sessions lasting for 30 min  **Response definition:** Improvement in pain severity based on VAS score of 25%, attack duration, patients’ quality of life (MSQ) and self-assessing questionnaires for depression and anxiety (SAS and SDS) | **Modality**: RS fMRI  **Time-points**: In patients the MRI was obtained before and after 4 weeks of treatment  HC underwent only one MRI | **Baseline: Patients vs HC**  ↑ Activation of the left thalamus, left inferior parietal gyrus, bilateral precentral gyrus, right postcentral gyrus, and bilateral supplementary motor area in patients compared to HC  ↓ Activation in the bilateral precuneus and left superior frontal gyrus/medial prefrontal cortex in patients compared to HC  **Baseline-to-follow-up:**  ↓ Activation in the left thalamus, bilateral prefrontal gyrus, right postcentral gyrus, bilateral supplementary motor area, left inferior parietal gyrus, bilateral precuneus in patients after treatment  **Clinical correlations**:  ↓ Activity of the right postcentral was negatively associated with the reduction in migraine pain severity after treatment  ↓ Activity of the bilateral precuneus was positively associated with the reduction in the migraine attack frequency after treatment | No sham device |
| Fu *et al.*, 2022^73^ | 70 MO  70 HC | **Type:** Real transcutaneous auricular vagus nerve stimulation (aVNS) applied at the left cymba concha and sham aVNS applied on the left tail of the helix  **Duration:** 4 weeks: 12 treatment sessions lasting for 30 min  **Response to treatment:** reduction of pain severity and migraine frequency | **Modality:** RS fMRI  **Time-points**: In patients the MRI was obtained before and after 4 weeks of treatment  HC underwent only one MRI | **Baseline: patients vs HC**  A model including the activity of the TCC/RVM, thalamus, medial prefrontal cortex, and temporal gyrus could discriminate migraine patients from controls with an accuracy of 79%, and could predict patients’ treatment response  **Baseline to follow-up:**  In migraine patients, changes in the activity of the right TCC/RVM, bilateral prefrontal cortex, right temporal gyrus, left insula, and left middle cingulate cortex after treatments  **Clinical correlation**:  ↓ Headache days after treatments had:  - Positive correlation with the activity of the TCC/RVM, bilateral prefrontal cortex, and left middle cingulate cortex  - Negative correlation with the activity of left insula and right temporal gyrus | No information regarding the direction of functional changes observed after treatment  External validation cohort is missing |
| Markin *et al.*, 2022^74^ | 19 episodic MO interictal (14 responders and 5 non-responders) | **Type:** Repetitive transcranial magnetic stimulation (rTMS)  **Duration**: Five rTMS sessions in 5 days during the headache-free period.  **Response to treatment**: Acute pain severity, frequency of headaches and presence of depression (HADS-depression tests) | **Modality:** RS fMRI (1.5 T)  **Time-points:** Before and after treatment | **Baseline-to-follow-up:**  ↑ FC of the visual areas, precuneus and precentral gyrus within the default mode network, as well as of the lateral occipital cortex within the visual network after treatment  ↓ FC of the postcentral gyrus and lateral occipital cortex within the salience network, as well as of the anterior cingulate cortex within the visual network after treatment  **Baseline-to-follow-up: Responder vs non-responders:**  ↑ FC of the medial prefrontal cortex within default mode network in non-responders compared to responders | No HC group  Small sample size |
| Schading *et al.*, 2021^75^ | 24 episodic Mx (11 verum and 13 sham tDCS)  31 HC | **Type:** Verum or sham occipital transcranial direct current stimulation (tDCS) applied over the visual cortex  **Duration**: Verum tDCS was performed daily for 20 min for 28 days.  Sham tDCS was performed daily for 30 seconds for 28days. | **Modality:** T1w images  **Time-points:** Patients were studied before treatment, after 1.5 months after treatment and 4 months of treatment suspension  HC were studied at baseline and 1.5 months post-baseline | **Baseline: patients vs HC**  ↑ GM volume of the left lingual gyrus in patients compared to controls  **Baseline-to-follow-up: Verum vs sham tDCS**  ↓ GM volume of the left lingual gyrus in patients after verum treatment compared to controls | Small sample size for subgroups of patients  Analysis was uncorrected for multiple comparisons |
| Matharu *et al.*, 2004^77^ | 8 CM | **Type:** Implanted bilateral greater occipital nerve stimulator  **Duration:** Stimulation settings were individually chosen by patients according to the pain relief  **Response to treatment:** relief of pain | **Modality:** PET  **Time-points**: Each patient had three PET scans: 1) stimulator at optimum settings and patient pain-free; 2) stimulator off and patient in pain; 3) stimulator partially activated and patient with intermediate levels of pain | **Correlation with pain**:  Significant association between changes in pain severity and changes in the CBF of the dorsal rostral pons, right basal ganglia, ACC, postcentral gyrus, cuneus, precuneus, cerebellum, frontal temporal, and occipital cortices in patients after treatment | No HC group  Small sample size  Analysis was uncorrected for multiple comparisons |

Abbreviations: ACC = Anterior cingulate cortex; CBF = Cerebral blood flow; CM = Chronic migraine; eTNS= Electrical external trigeminal neurostimulation; fMRI = functional magnetic resonance imaging; ^18^FDG = 18-fluorodeoxyglucose; GM= Grey matter; HADS= Hospital Anxiety and Depression Scale; HC = Healthy controls; MO= migraine without aura; MRI= magnetic resonance imaging; Mx= migraine with or without aura; MSQ= Migraine Specific Quality-of-Life Questionnaire; PET= positron emission tomography; RS= resting-state; rTMS = Repetitive transcranial magnetic stimulation; SAS= Self-Rating Anxiety Scale; SDS= Self-Rating Depression Scale; taVNS= transcutaneous auricular vagus nerve stimulation; RVM= Rostral ventromedial medulla; TCC = Trigeminocervical complex; T1w= T1-weighted.

**Supplementary Table 4. Behavioral approaches for migraine prophylaxis.**

| **Reference** | **Population** | **Treatment** | **Imaging modality** | **Results** | **Limitations** |
| --- | --- | --- | --- | --- | --- |
| Nahman-Averbuch *et al.*, Headache 2020^78^ | 18 Mx adolescents | **Type**: Cognitive behavioral therapy (CBT)  **Duration**: 8 weeks: weekly sessions lasting 45 min  **Response to treatment:** Reduction in pain intensity | **Modality:** RS fMRI and ASL  **Time-points**: Before and after treatment | **Baseline-to-follow-up:**  ASL results after treatment:   - ↑ rCBFof frontal brain areas - ↓ rCBF of the cerebellum   RS FC results after treatment:   - ↑ FC between the left amygdala and the bilateral paracingulate gyrus, dorsomedial and dorsolateral prefrontal cortex, right angular gyrus and lateral occipital cortex - ↑ FC between the right amygdala and the right lateral occipital cortex   **Clinical correlation**:  ↓ Headache days after treatment was associated with:  - ↑ rCBF of bilateral occipital and parietal brain areas  - ↑ FC of the left amygdala with the precuneus, lateral occipital cortex and cuneal cortex  - ↓ FC of the right amygdala with the paracingulate cortex and dorsolateral prefrontal cortex | No HC group  Small sample size |
| Nahman-Averbuch *et al.*,  Pain 2021^79^ | 19 Mx adolescents | **Type:** Cognitive behavioral therapy (CBT)  **Duration: 8 weeks:** weekly sessions lasting 45 min  **Response to treatment:** 50%  reduction in headache frequency | **Modality:** RS fMRI  **Time-points:** Before and after treatment | **Prediction of treatment response**  The FC of the right amygdala with the ACC, frontal and sensorimotor regions at baseline could predict headache days reduction after treatment | No HC group  Small sample size  Same cohort included in the study Nahman-Averbuch *et al.* 2020^84^ |
| Dobos *et al.*, 2021^80^ | 11 MO interictal  15 HC | **Type:** Autogenic training (AT)  **Duration:** 16 weeks: 50 min training sessions, weekly session  **Response to treatment**: Reduction of headache frequency | **Modality:** Task fMRI during the exposure of faces expressing four types of emotion: fear, happiness, sadness, and neutrality  **Time-points:** Before and after 16 weeks of treatment | **Baseline-to-follow-up: Patients vs HC**  ↓ Activity of the left medial frontal gyrus in response to fearful faces in migraine patients compared to controls  ↑ Activity of the left insula in response to happy faces in patients compared to controls  **Baseline-to-follow-up in patients:**  ↓ Activity of the dorsal pons in response to fearful faces after AT treatments | Small sample size |
| Seminowicz *et al.*, 2020^82^ | 98 Episodic Mx (50 MBSR+, 48 SMH) | **Type**: Enhanced mindfulness-based stress reduction (MBSR+) and stress management for headache (SMH)  **Duration**: 4 months: 12 sessions of about 2h, delivered weekly by group for 8 weeks, then biweekly for another 8 weeks  **Response to treatment:** Reduction in headache frequency | **Modality:** RS fMRI, task fMRI during painful thermal stimulation, fMRI during cognitive task and T1w MRI  **Time-points:** Before and after 20 weeks of treatment | **Baseline-to-follow-up: MBSR vs SMH**  ↓ Activity of bilateral cuneus and right parietal operculum in the MBSR+ group compared to the SMH group during the cognitive task  ↓ RS FC of the insula with the right posterior parietal cortex and right cuneus in the MBSR+ group compared to the SMH group  No significant between-treatment differences in changes of brain volumes or brain activation during painful stimulation | No HC group |

Abbreviations: ACC = Anterior cingulate cortex; AT= Autogenic training, CBT= Cognitive behavioral therapy; HC = Healthy controls; fMRI = functional magnetic resonance imaging; MBSR+= Enhanced mindfulness-based stress reduction program; MO= migraine without aura; MRI= magnetic resonance imaging; Mx= migraine with or without aura; rCBF = Regional cerebral blood flow; SMH= Stress Management for Headache.
